# Association of socio-economic position and suicide/attempted suicide in low and middle income countries in South and South-East Asia – a systematic review

**DOI:** 10.1186/s12889-015-2301-5

**Published:** 2015-10-15

**Authors:** Duleeka W. Knipe, Robert Carroll, Kyla H. Thomas, Anna Pease, David Gunnell, Chris Metcalfe

**Affiliations:** School of Social and Community Medicine, University of Bristol, Canynge Hall, 39 Whatley Road, Bristol, BS8 2PS UK; South Asian Clinical Toxicology Research Collaboration (SACTRC), Faculty of Medicine, University of Peradeniya, Peradeniya, Sri Lanka

**Keywords:** Suicide, Attempted suicide, Low and middle income, Asia, Socioeconomic position, Systematic review

## Abstract

**Background:**

Forty percent of the world’s suicide deaths occur in low and middle income countries (LAMIC) in Asia. There is a recognition that social factors, such as socioeconomic position (SEP), play an important role in determining suicidal risk in high income countries, but less is known about the association in LAMIC.

**Methods:**

The objective of this systematic review was to synthesise existing evidence of the association between SEP and attempted suicide/suicide risk in LAMIC countries in South and South East Asia. Web of Science, MEDLINE, MEDLINE in Process, EMBASE, PsycINFO, and article reference lists/forward citations were searched for eligible studies. Epidemiological studies reporting on the association of individual SEP with suicide and attempted suicide were included. Study quality was assessed using an adapted rating tool and a narrative synthesis was conducted.

**Results:**

Thirty-one studies from nine countries were identified; 31 different measures of SEP were reported, with education being the most frequently recorded. Most studies suggest that lower levels of SEP are associated with an increased risk of suicide/attempted suicide, though findings are not always consistent between and within countries. Over half of the studies included in this review were of moderate/low quality. The SEP risk factors with the most consistent association across studies were asset based measures (e.g. composite measures); education; measures of financial difficulty and subjective measures of financial circumstance. Several studies show a greater than threefold increased risk in lower SEP groups with the largest and most consistent association with subjective measures of financial circumstance.

**Conclusion:**

The current evidence suggests that lower SEP increases the likelihood of suicide/attempted suicide in LAMIC in South and South East Asia. However, the findings are severely limited by study quality; larger better quality studies are therefore needed.

**Systematic review registration:**

PROSPERO 2014:CRD42014006521

**Electronic supplementary material:**

The online version of this article (doi:10.1186/s12889-015-2301-5) contains supplementary material, which is available to authorized users.

## Background

Suicide is a major cause of premature mortality in low and middle income countries (LAMIC); 40 % of deaths occur in LAMIC in Asia [1]. There is a recognition that social factors, such as socioeconomic position (SEP), play an important role in determining suicide risk in high income countries [2, 3]. Evidence from a selective review suggested that low SEP is also associated with an increased risk of suicide in LAMIC [4], but only a few selected measures of SEP were investigated and a comprehensive search of the literature was not undertaken. Another review looking at only area level measures of SEP and suicide risk indicated that lower levels of area level SEP increased the risk of suicide in both high income and LAMIC [5]. Individual studies from LAMIC indicate both an increase and decreased risk of suicide and attempted suicide with lower SEP, with some studies reporting over a 3 fold increased odds in poorer groups [6–13].

The mechanism by which SEP impacts on the risk of suicide and suicide attempt is important to understand in order to better inform public health policy, and therefore plan more effective suicide prevention programs. It maybe that individuals with a lower SEP experience higher levels of adversity/stress and fewer life chances which could increase their susceptibility to mental illness [14] and psychological distress (e.g. feelings of hopelessness, entrapment)[15, 16], and therefore increase the risk of suicide. This may be further aggravated by the fact that individuals of lower SEP are less likely to have access or engage with health services. Conversely, it could be that poorer mental health impacts on an individual’s opportunity for upward social mobility or result in a fall in status, for example because of lower earnings/inability to work [14]. A further, more novel, mechanism could be that individuals with a lower SEP may be exposed to higher levels of environmental toxins (e.g. pesticides) which may impact on their propensity for engaging in impulsive behaviour and/or the prevalence of depression [17].

A systematic review of common mental disorders (excluding suicide and self-harm) in LAMIC concluded that most studies reported a positive association between poverty measures and common mental disorders, though different measures of poverty yielded different results [18]. This review included only a small proportion of longitudinal studies (10 %). It is important to note that not all suicide attempts in LAMIC in Asia have a preceding diagnosis of a mental health disorder [19, 20].

We conducted this review in order to systematically synthesise epidemiological evidence of the association between SEP and attempted suicide and suicide risk in LAMIC countries in South and South East Asia. The focus is on countries in South and South East Asia only in order to limit the cultural heterogeneity which would result from including all LAMIC countries.

## Methods

### Protocol and registration

Methods of the search strategy and inclusion criteria were specified in advance and documented in a protocol: PROSPERO 2014: CRD42014006521 (available from: http://www.crd.york.ac.uk/PROSPERO/display_record.asp?ID=CRD42014006521).

### Eligibility criteria

#### Types of studies

All epidemiological study designs on individual subjects were included (cross sectional studies, case–control, cohort and randomised control trials (results from control arm)). Qualitative studies, ecological studies, case series and reviews were excluded.

#### Types of participants

Human populations in LAMIC in South and South East Asia were included in this review [21]. The studies must have been carried out in a general population sample. We did not exclude studies based on age or gender. However, studies carried out in student populations were excluded as they were unlikely to be a representative sample of the total population; especially as access to and attendance at educational institutions is socially patterned and the inequality in access to education is most pronounced in LAMIC [22]. Likewise, studies comparing suicide attempts/deaths with other diseases/outcomes (e.g. accidents [23] or other deaths [20, 24]) which are likely to be socially patterned, were excluded as these may lead to underestimation of the association of SEP with suicide/attempted suicide risk.

#### Types of exposure measures

The exposure was individual SEP (i.e. not area level) and was defined to include both social and economic factors that are important in determining a person’s place in society. Hence we considered the following SEP measures:

1. SEP- aggregate/composite measure (a measure which gives an overall picture of a person’s SEP; this can include several measures of SEP – see Additional file 1)
2. Social class/caste
3. Education level
4. Occupation
5. Income level
6. Marital status
7. Religion
8. Social position (a measure of position in society)
9. Vehicle ownership
10. Household construction
11. Cooking fuel/electricity supply (access to services)

Marital status was included as a measure of SEP, because in an Asian context being married (especially for women) confers a certain status within the community. In particular older unmarried women are stigmatised in certain countries in Asia [25]. In addition, individuals who are unmarried because they are divorced/widowed/separated, may have a lower status within society and be economically disadvantaged due to property laws - this is particularly pronounced in women.

Religion was also included as a measure of SEP because in Asia, particularly South Asia, being religious is the norm [26] and not being religious is a minority status. This minority status may therefore lead to a diminished status within society. In addition, having a minority faith may also confer a lower status within the community the person lives in, for example a Muslim living in a predominantly Buddhist society.

#### Types of outcome measures

The primary outcome measures were suicide and suicide attempts.

#### Report characteristics

No date or language restrictions were applied to publications. Non-English papers were screened and extracted with the help of a native speaker. All studies, regardless of publication status were eligible to be included in this review. If a single study was reported in multiple reports, the report with the most comprehensive data was used to extract data and the other reports used to supplement this.

### Information sources

We conducted searches of computer databases as follows: Web of Science, MEDLINE (1950 onwards), MEDLINE in Process, EMBASE and PsycINFO. The search strategy is included in the supplementary material (see Additional file 1). Search terms were mapped to MeSH terms/subject headings and in keyword searches. Reference searches were conducted on all included papers, and forward citations on key papers and reviews [20, 24, 27–29]. A selection of experts (one from each country included in the review if one was available) were sent a list of all papers identified for inclusion in the review and asked to review these and highlight any papers missed. The last search was run on 20^th^ May 2013. For papers where data were not presented for the association of the exposure (SEP) and outcome, we contacted authors for these data if the study was published in the last 5 years. Of those contacted 24 % (*n* = 4/16) responded, though only one study was able to provide the data requested.

### Study selection

Eligibility assessment of titles and abstracts were performed by 2 independent reviewers and disagreements checked by a third independent reviewer. Full texts of all potentially eligible studies were obtained and these were then screened for eligibility by two independent reviewers and discrepancies resolved by consensus. In addition, in recognition that SEP is routinely collected in studies but often will not appear as a result in the abstract, DK screened the full text of all papers which would have been excluded in the title/abstract screen as not having a measure of SEP but would have otherwise been eligible.

### Data extraction

Using a piloted data extraction sheet two review authors independently extracted data on study design, study participants, exposure and outcome details, and measures of associations of SEP and suicide/attempted suicide. For multi-country papers where the data was extractable for each country separately, each country was included as a separate study. There were 3 multi-country publications [30–32], resulting in 7 studies being included in the review.

#### Quality assessment

We assessed the quality of publications included in this review for risk of bias, by using the Newcastle-Ottawa scale for assessing the quality of non-randomised studies [33]. The assessment was done at the study level. This scale utilises a star system which judges the quality of a paper on three broad areas: participant selection; comparability of groups; and exposure/outcome ascertainment (depending on study design). The scale is not comparable between study designs and so can only assess the quality of the study compared to other studies of a similar design. We used an adapted version of this scale in order for the assessment criteria to be relevant to the exposure and outcomes of interest (see Additional file 1). Two independent reviewers assessed all included English language papers and the lead author, with the aid of a translator, assessed the quality of the non-English papers. For each study type we used the middle quality rating (≥50 %) to categorise studies as reasonable/high quality. These papers were used in order to conclude the direction of effect of each SEP measure and suicide/attempted suicide.

### Analysis

All data extracted were entered into a Microsoft Access database. Studies were described according to the country of data collection, sampling frame, sample size, response rate, sex, age, the type of outcome and outcome ascertainment. Studies were assumed to include all ages if not otherwise stated.

The studies included in this review were very heterogeneous. Studies differed in terms of the outcome measured (i.e. suicide vs. suicide attempts), SEP measures, and study population; nine different countries were included in this review, and the mechanism and extent in which low SEP impacts on suicide/attempted suicide risk is likely to be different by country. Due to these clinical and methodological differences we decided that it would be inappropriate to conduct a meta-analysis. There were 31 different SEP measures reported. In order to present these results we grouped these SEP measures into major and minor categories (see Additional file 1) and have used this to structure the review. We only present the conventional measures of SEP in the main text, but the less-conventional measures (marital status and religion) are provided in the supplementary results (see Additional file 2). Using the *metan* command in Stata (version 12) we generated forest plots without the pooled estimates for each SEP minor grouping category. If a study presented both an adjusted and unadjusted effect estimate, we used the fully adjusted estimate when creating the forest plots. We categorised studies according to what was adjusted for in the analysis for each SEP measure presented (see Additional file 1).

For matched case control studies it was not always clear if a matched analysis was conducted, nor was it possible to calculate ORs accounting for the matching. The use of unadjusted analysis in matched studies leads to inconsistent findings [34]. We used the findings from the high quality studies (quality score ≥50) and studies which used an appropriate analysis for a matched case control study to assess whether the exclusion of the lower quality studies affected the findings. Taking a conservative approach, we categorised studies which didn’t explicitly mention a matched analysis, as not having conducted one.

## Results

### Study characteristics

**Fig. 1:**
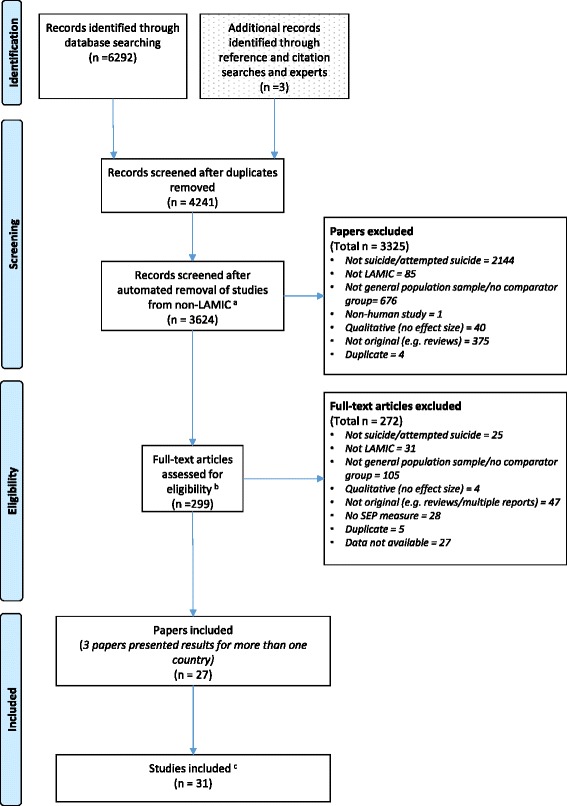
Flow chart showing the selection of papers for inclusion in the systematic review. ^a^Records screened by two study authors. ^b^142 articles not meeting the inclusion criteria based on the title/abstract screen were checked for eligibility by two study authors. An additional eligibility check was done on 157 papers by a single study author. ^c^There were 3 multi-country publications [30–32], resulting in 7 studies

After removal of duplicates the database search identified 4238 records for title and abstract screening (Fig. 1). The titles, abstracts and full-text articles were screened to assess whether they met the eligibility criteria outlined in the methods section. A total of 28 (9 %) papers indicated in the full-text that they had recorded SEP as part of their study but did not report on it. We identified 27 papers to be included for this review [6–13, 30–32, 35–50]; three of these were multi-country papers reporting on 7 country-specific effect estimates of an association of suicide/attempted suicide with SEP. This resulted in 31 different studies being included in this review.

Study characteristics are described in Table 1 (cross-sectional and cohort studies) and Table 2 (case–control studies). The majority of studies included were from China (56 %) and India (29 %), with 2 studies each from Thailand and Vietnam, and only one study each from Bangladesh, Indonesia, Pakistan, Philippines and Sri Lanka. Case–control studies were the most popular study design (48 %), only 3 (10 %) relevant cohort studies were identified. The majority of studies (55 %) reported on attempted suicide only, with 10 % reporting on both attempted suicide and suicide. Just over half of the studies used self-reported outcomes. Seven of the studies (23 %) (2 from the same paper) were conducted in a restricted age population (age range of 30 years or less) [6, 13, 30, 36, 40, 43] and three of the studies were conducted in women only (2 from the same paper) [31, 43] (Tables 1 and 2). The most commonly recorded SEP measure was education (71 %), followed by marital status (61 %) and occupation (52 %). It is important to note that not all recorded SEP measures were always reported or available through the study authors (Table 3). Studies reporting on both attempted suicide and suicide (combined outcomes) reported on all the measures of SEP included; whereas studies reporting on outcomes separately (suicide only or attempted suicide only) reported on a small range of SEP measures.

**Table 1:** Study characteristics of cross-sectional and cohort studies included (*n* = 16)

Study	Country	Study design	Sampling frame	Sample (Response rate %)	% Male	Age range	Outcome	Outcome ascertainment (time period)
Feroz (2012) [7]	Bangladesh	Cross-sectional	2008 National ID data	12422 (80 %)	52	All ages	Suicide & Attempted suicide	Self-report - household informant (ever)
Lee (2007) [39]	China	Cross-sectional	Census	1628 (74 %)	NR	18-70	Attempted suicide	Self-report (ever)
Ma (2009) [12]	China	Cross-sectional	Census	5926 (94 %)	46	≥15	Attempted suicide	Self-report (ever)
Sun (2010)[47]	China	Cross-sectional	Census	20716 (86 %)	50	≥18	Attempted suicide	Self-report (ever & last year)
Ma (2010) [41]	China	Cross-sectional	Geographical location	6625 (99 %)	48	15-69	Attempted suicide	Self-report (last year)
Dai (2011) [36]	China	Cross-sectional	Census	1654 (55 %)	47	16-34	Attempted suicide	Self-report (ever & last year)
Li (2011) [40]	China	Cross-sectional	NR	1032 (86 %)	52	≥60	Attempted suicide	Self-report (last year)
Chiu (2012) [6]	China	Cross-sectional	Census	263 (64 %)	52	≥50	Attempted suicide	Self-report (ever)
Blum (2012) – A [30]	China	Cross-sectional	Census	6212 (NR)	50	15-24	Attempted suicide	Self-report (last year)
Rebholz (2011) [44]	China	Cohort	Sampling centres	169871 (NR)	49	≥40	Suicide	Hospital records/death certificates (8.3 years mean follow-up)
Chowdury (2005) [35]	India	Cross-sectional	Geographical location	938 (100 %)	56	All ages	Suicide & Attempted suicide	Self-report - household informant (ever)
Maselko (2008) [43]	India	Cohort	Primary Care	2494 (83 %)	0	18-45	Suicide & Attempted suicide	Self-report (last year)
Sauvaget (2009) [45]	India	Cohort	Nested in an oral cancer screening trial	151728 (87 %)	38	≥35	Suicide	Death records (7.5 years mean follow-up)
Devries (2011) - A [31]	Thailand – City	Cross-sectional	Census	1379 (85 %)	0	15-49	Attempted suicide	Self-report (ever)
Devries (2011) - B [31]	Thailand – Province	Cross-sectional	Census	1140 (85 %)	0	15-49	Attempted suicide	Self-report (ever)
Blum (2012) - B [30]	Vietnam	Cross-sectional	Census	6191 (NR)	47	15-24	Attempted suicide	Self-report (last year)

NR - Not reported

**Table 2:** Study characteristics of case–control studies included (*n* = 15)

Study	Country	Cases N (response rate %) Sampling frame	Controls N (response rate %) Sampling frame	If matched – matching criteria	% Male	Age range	Outcome
Zhang (2004) [50]	China	66 (100 %) Public health personnel and villagers	66 (97 %) Neighbourhood	Age & sex	73	All ages	Suicide
Jia (2005) [9]	China	205 (NR) Hospital	205 (NR) Hospital and community	Age, sex & area	43	All ages	Attempted suicide
Zhang (2010) [13]	China	392 (98 %) Surveillance system	416 (97 %) Community controls		51	15-34	Suicide
Sun (2014) [46]	China	199 (91 %) Surveillance system	199 (NR) Unclear	Age, sex & residence	58	All ages	Suicide
Manoranjitham (2010) [42]	India	100 (92 %) Surveillance system	100 (100 %) Neighbourhood	Age, sex & neighbourhood	59	All ages	Suicide
Vijayakumar (1999) [49]	India	100 (81 %) Police	100 (94 %) Neighbourhood	Age, sex & neighbourhood	55	≥15	Suicide
Gururaj (2004) [8]	India	269 (NR) Police	269 (NR) Neighbourhood	Age, sex & community	64	All ages	Suicide
Sisask (2010) - A [32]	India	680 (NR) Hospital	500 (NR) Community		NR	All ages	Attempted suicide
Kulkarni (2011) [38]	India	100 (NR) NR	100 (NR) NR	Age & sex	63	All ages	Attempted suicide
Kumar (2013) [48]	India	50 (NR) Hospital	50 (NR) Community	Age, sex & marital status	44	≥18	Attempted suicide
Kurihara (2009) [11]	Indonesia	60 (94 %) Police	120 (100 %) Neighbourhood	Age & sex	NR	All ages	Suicide
Khan (2008) [10]	Pakistan	100 (NR) Police	100 (NR) Community	Age, sex & area of residence	83	All ages	Suicide
Jollant (2014) [37]	Philippines	15 (94 %) Local informants	30 (NR) Community	Age and sex	73	All ages	Suicide
Sisask (2010) - B [32]	Sri Lanka	300 (NR) Hospital	684 (NR) Community		NR	All ages	Attempted suicide
Sisask (2010) - C [32]	Vietnam	143 (NR) Hospital	2280 (NR) Community		NR	All ages	Attempted suicide

NR - Not reported

**Table 3:**
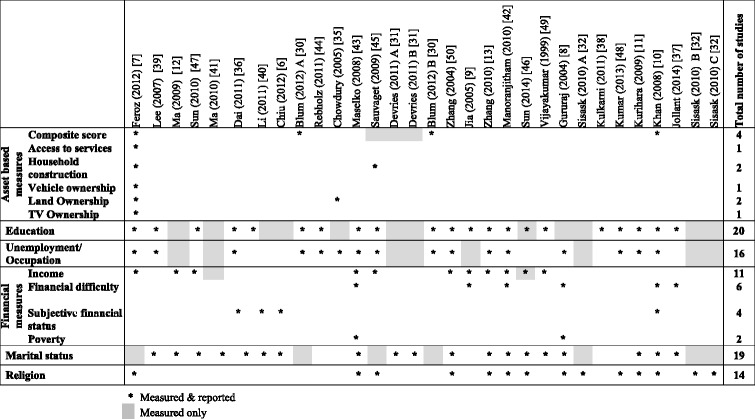
SEP measures reported/recorded by each included study

ᅟ

The detailed quality rating of the papers included can be found in the supplementary materials (see Additional file 2). None of the studies scored 100 %, with 8 studies scoring less than 50 % (2 from the same paper) [7, 12, 30, 38, 39, 43, 50].

### Associations of SEP with suicide/attempted suicide

#### Asset based measures

**Fig. 2:**
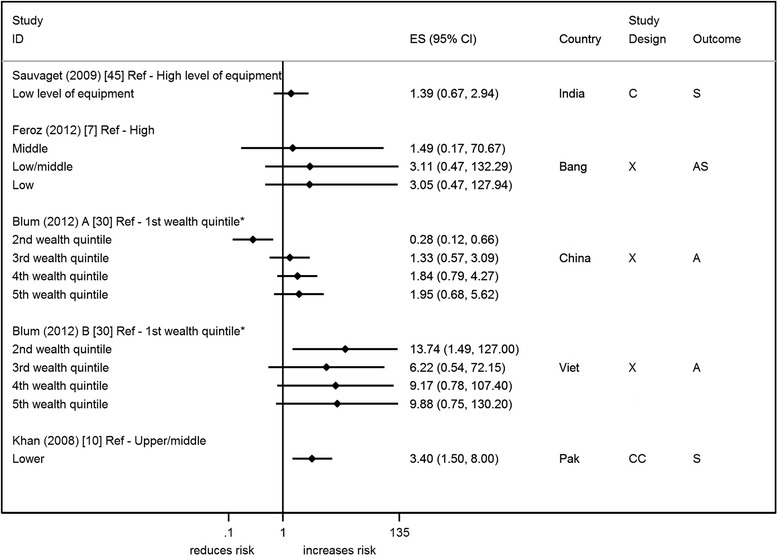
Forest plots of studies reporting on composite measures of SEP and suicide/attempted suicide risk. Country: Bang – Bangladesh; Indon – Indonesia; Pak –Pakistan; Phil – Philippines; SL – Sri Lanka; Thai – Thailand; Viet – Vietnam Study design: C – Cohort; CC – Case-Control; X – Cross-sectional Outcome: A – Attempted suicide only; S – Suicide only; AS – Attempted suicide and suicide ES: Cohort studies report estimates of relative risk (except for Maselko (2008) which reports odds ratios), and case-control/cross-sectional studies report odds ratios. *Highest wealth quintile

**Fig. 3:**
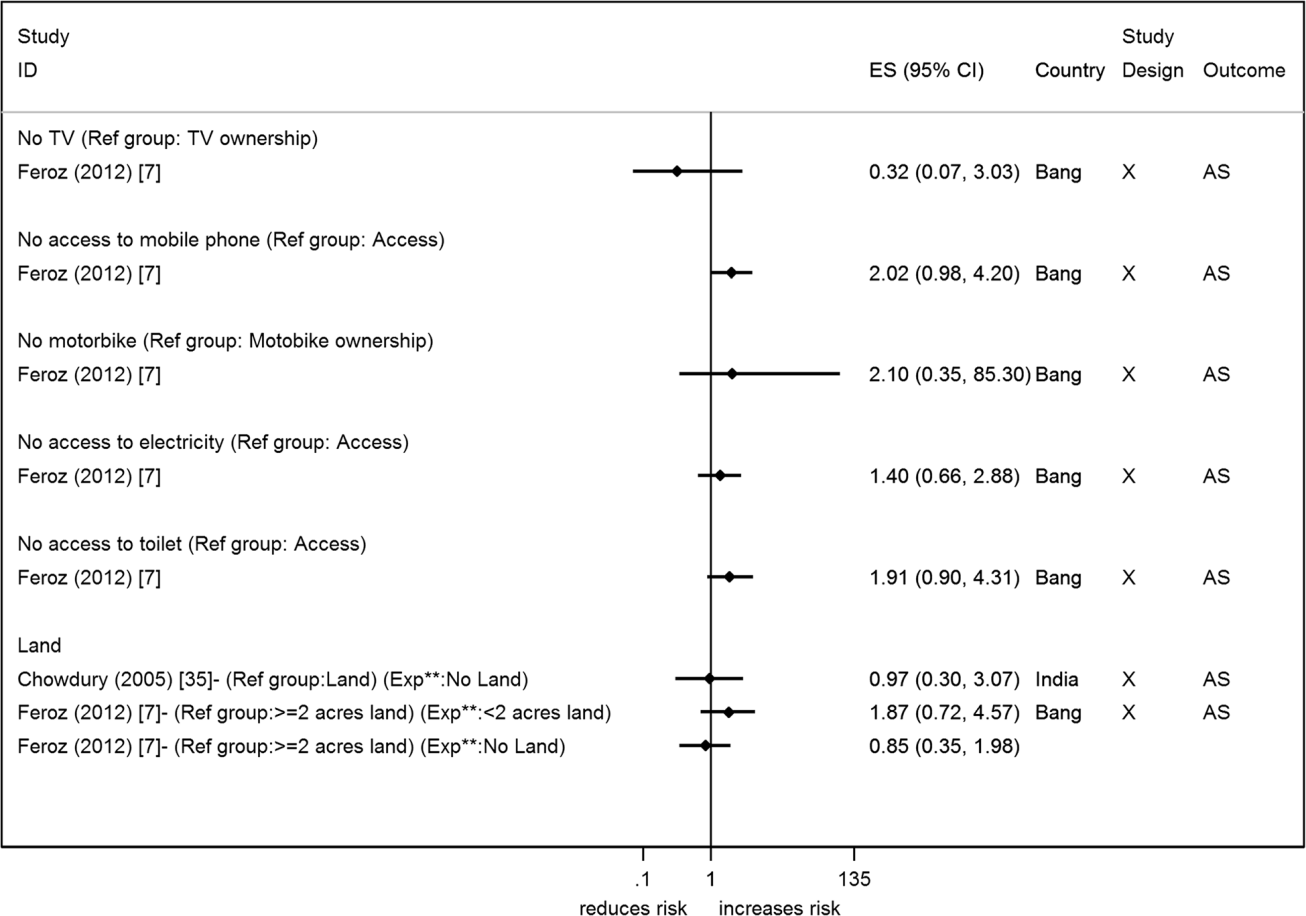
Forest plots of studies reporting on access to or ownership of assets and suicide/attempted suicide risk. Country: Bang – Bangladesh; Indon – Indonesia; Pak –Pakistan; Phil – Philippines; SL – Sri Lanka; Thai – Thailand; Viet – Vietnam Study design: C – Cohort; CC – Case-Control; X – Cross-sectional Outcome: A – Attempted suicide only; S – Suicide only; AS – Attempted suicide and suicide ES: Cohort studies report estimates of relative risk (except for Maselko (2008) which reports odds ratios), and case-control/cross-sectional studies report odds ratios. **Exposure group

**Fig. 4:**
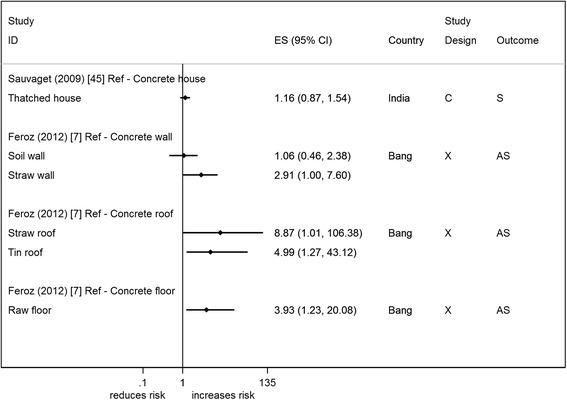
Forest plots of studies reporting on household construction and suicide/attempted suicide risk. Country: Bang – Bangladesh; Indon – Indonesia; Pak –Pakistan; Phil – Philippines; SL – Sri Lanka; Thai – Thailand; Viet – Vietnam Study design: C – Cohort; CC – Case-Control; X – Cross-sectional Outcome: A – Attempted suicide only; S – Suicide only; AS – Attempted suicide and suicide ES: Cohort studies report estimates of relative risk (except for Maselko (2008) which reports odds ratios), and case-control/cross-sectional studies report odds ratios

Asset based measures included composite measures (aggregate scores), asset ownership/access and quality of household construction. Only 6 studies reported on an asset based measure of SEP (2 from the same paper) [7, 10, 30, 35, 45] (Figs. 2, 3 and 4 ). This is despite the recognition that asset based measures are, in LAMIC, one of the most robust measures of SEP [22]. The asset based measures can be broadly categorised as: composite measures; access/ownership of assets, e.g. vehicle ownership; and household construction. Five studies used a composite measure of SEP (2 of the studies are from the same paper) [7, 10, 30, 45] (Fig. 2). The way in which these composite scores were derived varies from study to study and can include income, ownership of assets, access to services (e.g. electricity, mains water), education, and occupation (see Additional file 2). Therefore the comparability of these measures between studies is limited.

Most studies show an increased risk of suicide/attempted suicide in individuals with a composite score indicating fewer assets [7, 10, 30, 45], though only one study from Pakistan has shown statistical evidence to support this (OR 3.4, 95 % CI 1.50, 8.00) [10] (Fig. 2). Studies that have more than one level of composite SEP [7, 10, 30] show no clear indication of a trend. Three of the studies [10, 30, 45] have made adjustments in the design or in the analysis for other factors (see Additional file 2). Some of these factors are also alternative measures of SEP (e.g. area of residence, education), so adjusting for them may be considered inappropriate (over-adjustment). Only one study presented an adjusted and unadjusted estimate. Adjusting for age, gender, SEP and several other factors reduced the effect estimate by 35 % from 2.13 (95 % CI 1.05, 4.35) to 1.39 (95 % CI 1.05, 4.35) [45]. The restricted analysis (which included studies with a reasonable/high quality score and only appropriately analysed matched case control studies) included only two studies [10, 45]; both studies are consistent with an increased risk of suicide with poorer composite score.

Only two cross-sectional household surveys report on asset ownership or access to assets [7, 35], both in relation to attempted suicide and suicide. Both studies report on land ownership but there is no clear indication that lack of ownership increases the odds of suicide/attempted suicide (Fig. 3). Ownership/access to a motorbike, toilet, mobile phone, and electricity were all measured in the same study and indicated that lack of these items increased the odds of suicide/attempted suicide, though there was no/weak statistical evidence for these associations [7]. Both studies have self-reported outcomes and make no adjustments for any other factors. Only one study was of reasonable/high quality and it failed to show an association of land ownership with suicide/attempted suicide [35].

Two studies from India (cohort) and Bangladesh (cross-sectional) measured the quality of the materials used in the construction of the house [7, 45] (Fig. 4). House construction is said to give an idea of accumulated wealth of a household. The studies that report on house construction indicate that poorer quality construction materials increase the odds of suicide/attempted suicide, though this association was not supported by the statistical evidence. The cohort study (*n* = 151,728) provided an adjusted and unadjusted estimate [45]; the crude estimate for this study provides some statistical evidence that poor house construction is associated with suicide (RR 2.13 95 % CI 1.05, 4.35), but once adjustments were made (including other SEP measures) the effect estimate was attenuated towards the null (RR 1.16 95 % CI 0.87,1.54)) [45]. This study was also the only one to be included in the restricted analysis with reasonable/high quality studies.

#### Education

Education level was reported in 20 studies (2 from the same paper) [7, 9–11, 13, 30, 36–40, 42–44, 47–50]. Education was recorded as the number of years completed (continuous and ordinal categorical) and highest qualification reached. One case–control study from India did not report an effect estimate nor did it describe how education level was measured [38]. This study reported that cases were significantly more likely to have lower levels of education than controls but did not report effect estimates. For another cross-sectional study from China we were unable to calculate an odds ratio for the association of no education with attempted suicide, as all (*n* = 8, 100 %) attempters were uneducated [40].

**Fig. 5:**
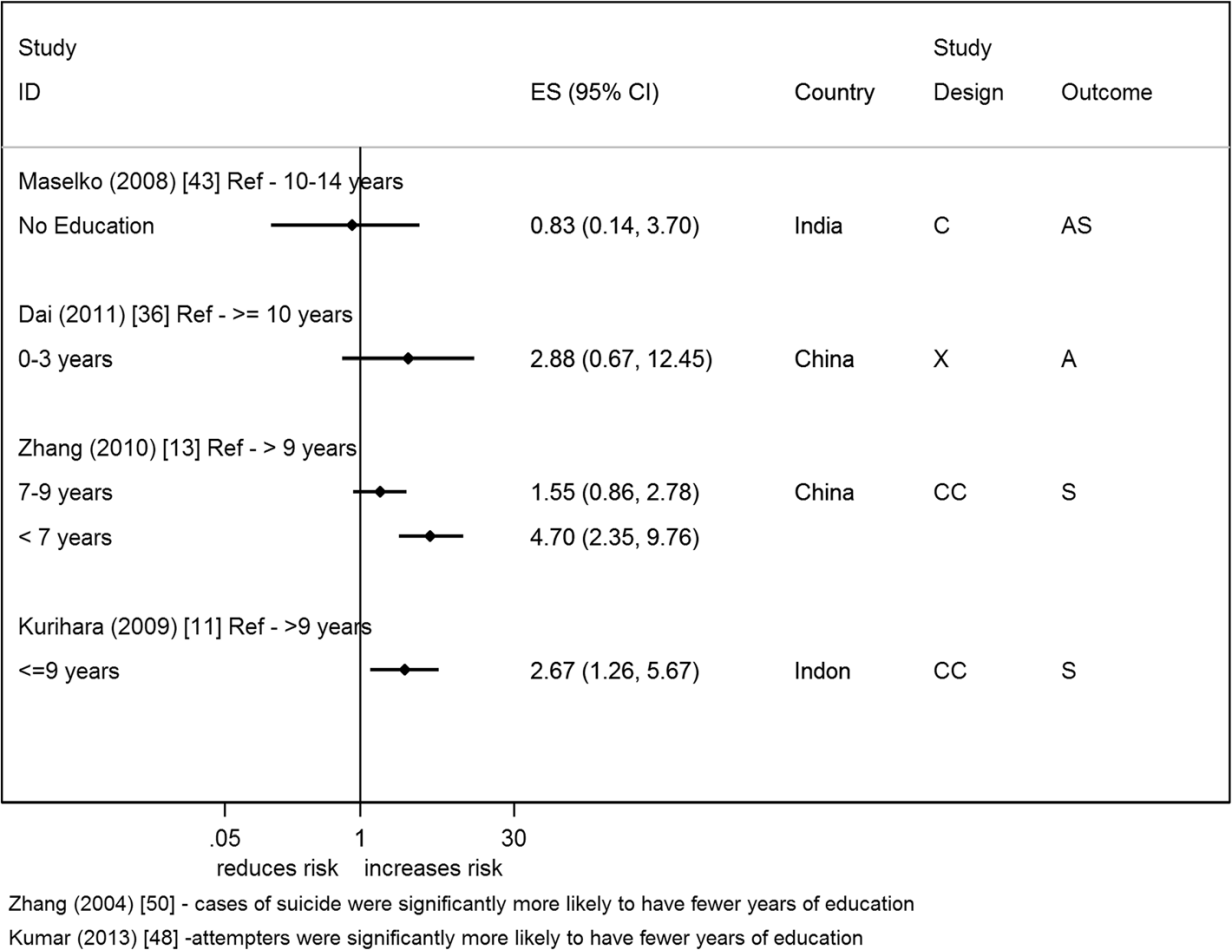
Forest plot of studies reporting on number of years of education and suicide/attempted suicide risk. Country: Bang – Bangladesh; Indon – Indonesia; Pak –Pakistan; Phil – Philippines; SL – Sri Lanka; Thai – Thailand; Viet – Vietnam. Study design: C – Cohort; CC – Case– control; X – Cross-sectional. Outcome: A – Attempted suicide only; S – Suicide only; AS – Attempted suicide and suicide. ES: Cohort studies report estimates of relative risk (except for Maselko (2008) which reports odds ratios), and case–control/cross-sectional studies report odds ratios

**Fig. 6:**
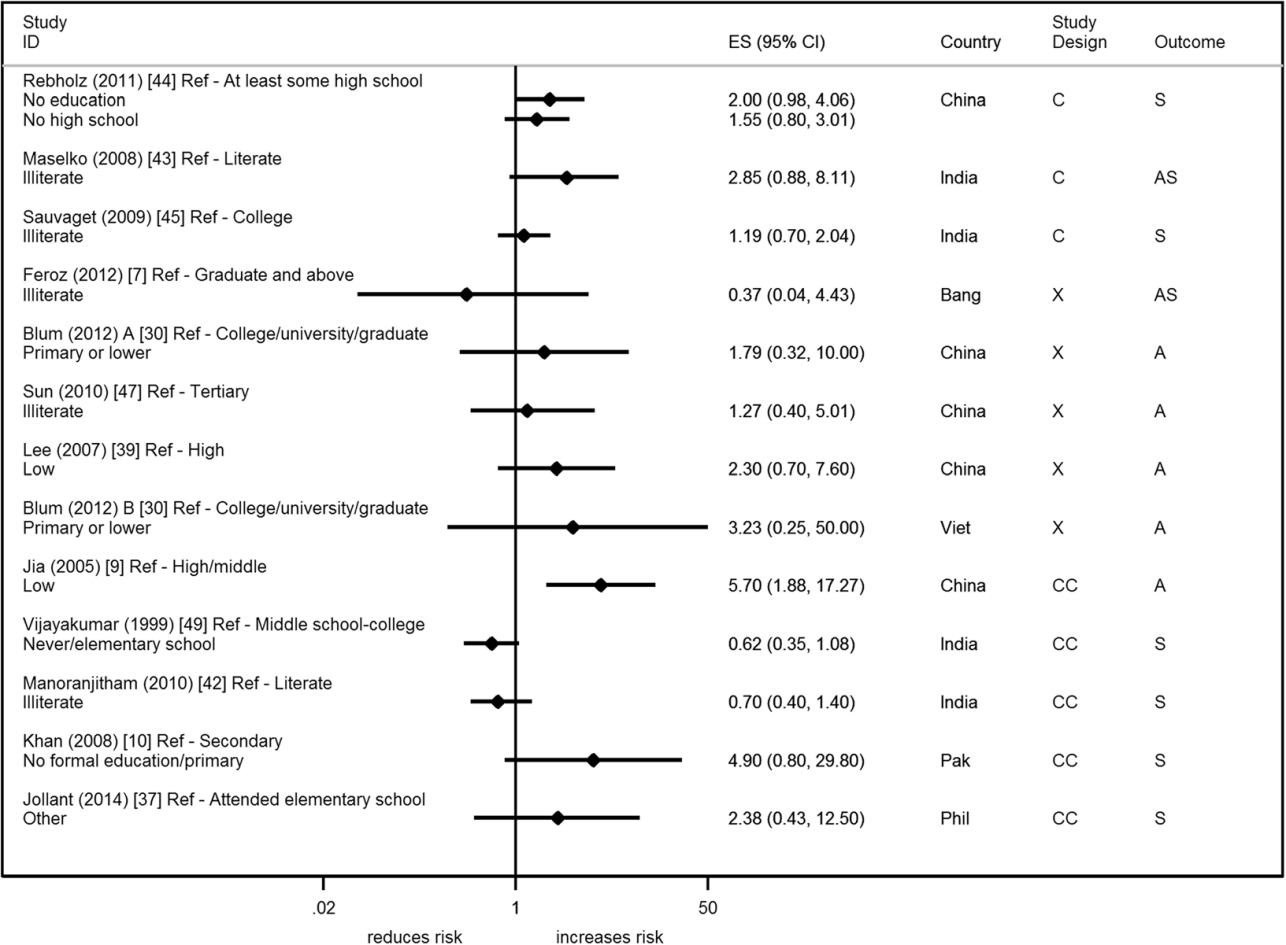
Forest plot of studies reporting on highest educational qualification and suicide/attempted suicide risk. Country: Bang – Bangladesh; Indon – Indonesia; Pak –Pakistan; Phil – Philippines; SL – Sri Lanka; Thai – Thailand; Viet – Vietnam. Study design: C – Cohort; CC – Case– control; X – Cross-sectional. Outcome: A – Attempted suicide only; S – Suicide only; AS – Attempted suicide and suicide. ES: Cohort studies report estimates of relative risk (except for Maselko (2008) which reports odds ratios), and case–control/cross-sectional studies report odds ratios

Six studies reported on years of education [11, 13, 36, 43, 48, 50] (Fig. 5). Most studies reported that fewer years of education were associated with an increased risk of suicide/attempted suicide [11, 13, 36, 48, 50] – OR ranged from 2.67 to 4.70. Thirteen studies (2 from the same paper) reported on education in terms of a person’s or head of household’s highest education qualification or literacy level [7, 9, 10, 30, 37, 39, 42–45, 47, 49] (Fig. 6). Studies generally showed no clear evidence of an association of suicide/attempted suicide with poorer educational qualifications or literacy, though one study showed a statistical evidence of an increased risk [9] (Fig. 6). Twelve studies presented effect estimates adjusted for a range of different factors [9–11, 13, 30, 36, 39, 42, 44, 45, 49]; four studies presented crude estimates [9, 10, 36, 45]. All but one study showed that the adjustments (which included other SEP factors) resulted in an increase in the effect size (away from the null). All these studies reported on the highest qualification. The study that didn’t show this increase showed a halving of the effect estimate; this study reported on years of education [36].

Eight studies were rated reasonable/high quality (36 %) [10, 13, 36, 42, 44, 45, 47, 48], and almost all these studies found that lower levels of education increases risk of suicide/attempted suicide. The one study which showed an opposite direction of effect was the only study to use literate individuals as the base category; all other studies used a higher education category as the comparison group (e.g. college graduates).

#### Occupation

Studies reported on a range of different occupation categorisations. These are split into studies investigating the effect of: unemployment, and different occupation types.

### Unemployment

**Fig. 7:**
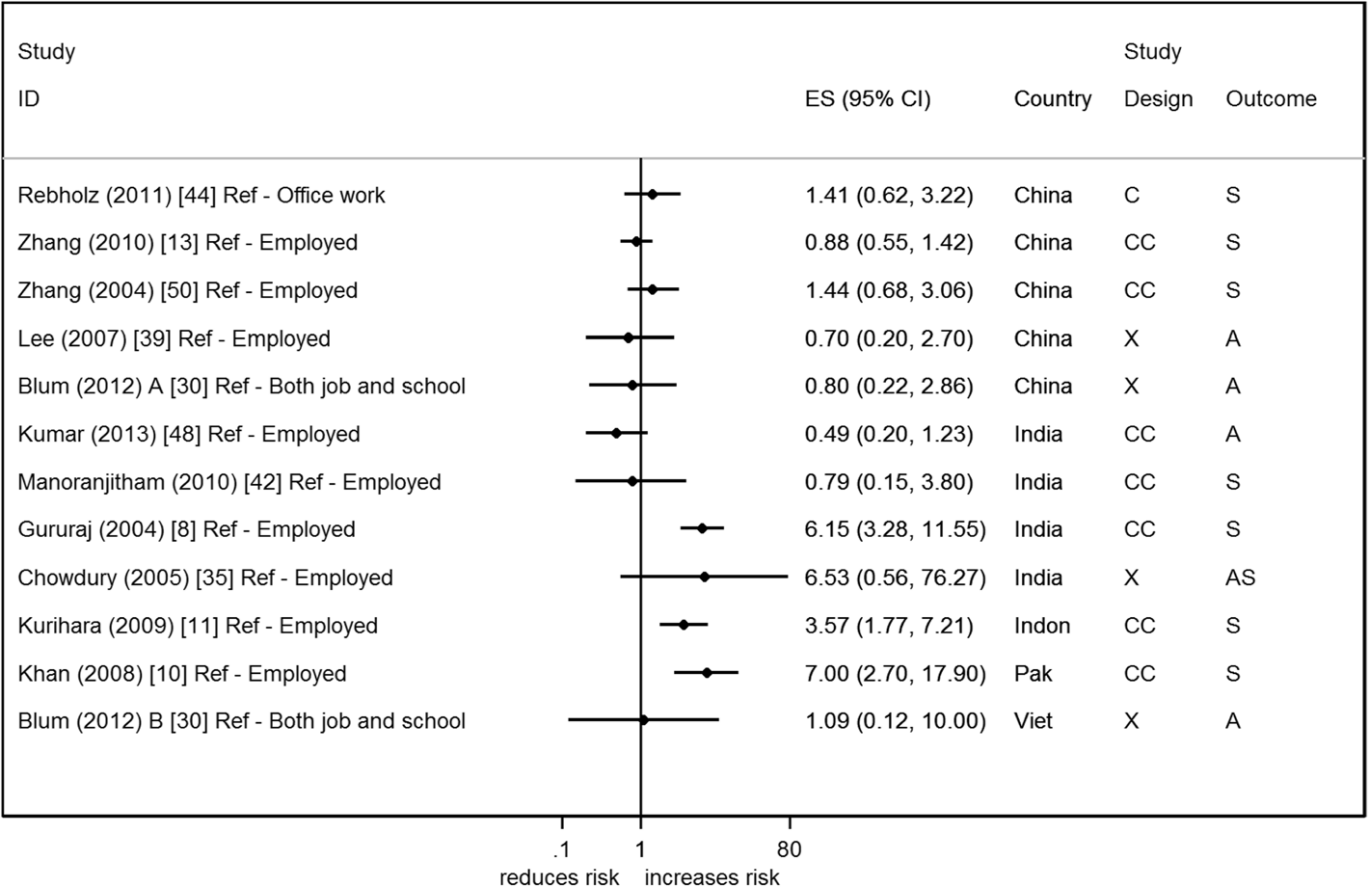
Forest plot of studies reporting on unemployment and suicide/attempted suicide risk. Country: Bang – Bangladesh; Indon – Indonesia; Pak –Pakistan; Phil – Philippines; SL – Sri Lanka; Thai – Thailand; Viet – Vietnam. Study design: C – Cohort; CC – Case–control; X – Cross-sectional. Outcome: A – Attempted suicide only; S – Suicide only; AS – Attempted suicide and suicide. ES: Cohort studies report estimates of relative risk (except for Maselko (2008) which reports odds ratios), and case–control/cross-sectional studies report odds ratios

Twelve studies reported on the association of unemployment with suicide/attempted suicide (2 from the same paper) [8, 10, 11, 13, 30, 35, 39, 42, 44, 48, 50] (Fig. 7). There was wide variability in effect estimates across studies. One of the problems with assimilating the results from these studies is that the definition of unemployment used is unclear. None of the studies explicitly describe who was classed as unemployed or the reference period that the unemployment refers to. Most of the study estimates are centred around the null, however findings from India, Indonesia and Pakistan (all in relation to suicide) show evidence of an increased risk in individuals who were unemployed (OR range from 3–7).

Seven of the studies controlled for other factors, with 6 of the studies making adjustments for other SEP factors [10, 30, 39, 42, 44]. Only one of the studies provided unadjusted estimates; Zhang *et al.* (2010) adjusted for age/gender and this resulted in a change in the point estimate from 1.02 (95 % CI 0.76, 1.39) to 0.88 (95 % CI 0.55, 1.42) [13].

When restricting the analysis to the reasonable/high quality studies [10, 13, 35, 42, 44], there was no association of unemployment with suicide/attempted suicide risk in most studies, though all of these studies adjusted for SEP in their analysis. Two studies, however, indicated an increased risk of suicide/attempted suicide with unemployment [10, 35]. One of the studies [35] used the unemployment status of the head of household to investigate this association and not individual unemployment. The other study was the only study to indicate that they excluded students and those with long term illness from their unemployed category [10].

### Occupation types

**Fig. 8:**
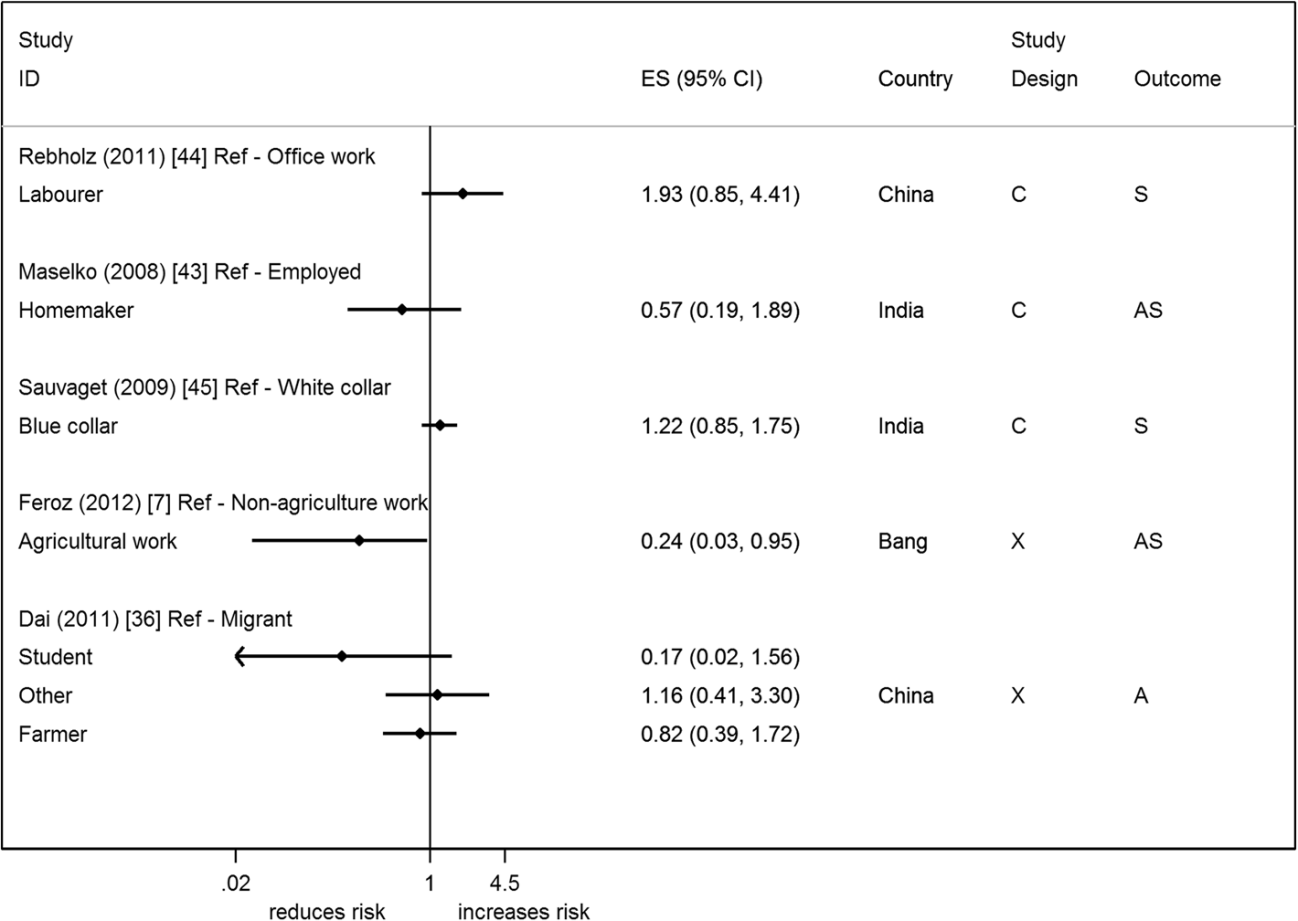
Forest plot of studies reporting on different occupation types and suicide/attempted suicide risk. Country: Bang – Bangladesh; Indon – Indonesia; Pak –Pakistan; Phil – Philippines; SL – Sri Lanka; Thai – Thailand; Viet – Vietnam. Study design: C – Cohort; CC – Case–control; X – Cross-sectional. Outcome: A – Attempted suicide only; S – Suicide only; AS – Attempted suicide and suicide. ES: Cohort studies report estimates of relative risk (except for Maselko (2008) which reports odds ratios), and case–control/cross-sectional studies report odds ratios

Five studies reported on occupation types and attempted suicide/suicide risk [7, 36, 43–45] (Fig. 8). The main comparison was between labour based occupations vs. non-labour occupations (e.g. professional/office based). Only one study showed weak statistical evidence of an association of a reduced risk of suicide/attempted suicide in agricultural workers vs. other in Bangladesh [7]. Three studies make adjustments for other factors (including other SEP indicators) which results in an attenuation of the effect estimate towards the null [36, 44, 45]. The reasonable/high quality studies [36, 44, 45] suggested an increased risk of suicide in labourers compared to non-labourers [44, 45].

#### Financial measures

Studies reported on a range of financial measures and were categorised into those using: estimated income; measures of financial difficulty; and subjective measures of financial circumstances.

### Income

**Fig. 9:**
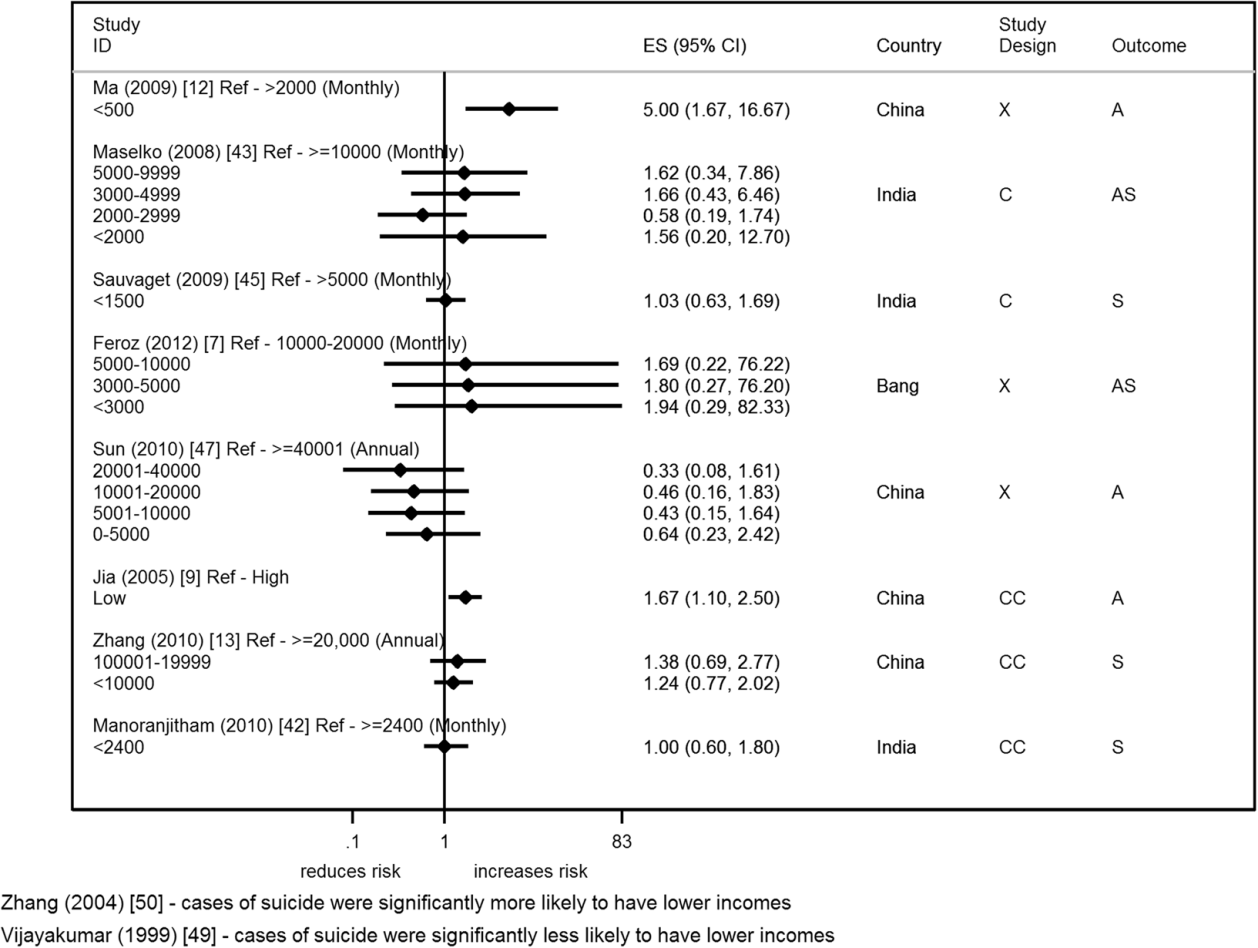
Forest plot of studies reporting on income and suicide/attempted suicide risk. Country: Bang – Bangladesh; Indon – Indonesia; Pak –Pakistan; Phil – Philippines; SL – Sri Lanka; Thai – Thailand; Viet – Vietnam. Study design: C – Cohort; CC – Case–control; X – Cross-sectional. Outcome: A – Attempted suicide only; S – Suicide only; AS – Attempted suicide and suicide. ES: Cohort studies report estimates of relative risk (except for Maselko (2008) which reports odds ratios), and case–control/cross-sectional studies report odds ratios

Ten studies reported on the association of attempted suicide/suicide with income level (Fig. 9) [7, 9, 12, 13, 42, 43, 45, 47, 49, 50]. Most studies showed an increased risk of suicide/attempted suicide with lower levels of income [7, 9, 12, 13, 50], with two studies reporting no difference [42, 45]. Out of those studies showing an increased risk, only two studies (a case control and a cross-sectional study) reported statistical evidence of an increased risk in low income individuals [9, 12]. Whereas a study from India showed statistical evidence of an opposite effect (continuous score) [49]. Four of the studies made adjustments in their analysis for other factors [12, 13, 42, 45] and all but one showed an attenuation of the effect towards the null after adjustment [13, 45]. This single study, which did not show this attenuation, was the only study not to adjust for other SEP factors [12]. Studies of reasonable/high quality included in the restricted analysis [13, 42, 45, 47, 49] continued to show mixed results.

### Financial difficulty

**Fig. 10:**
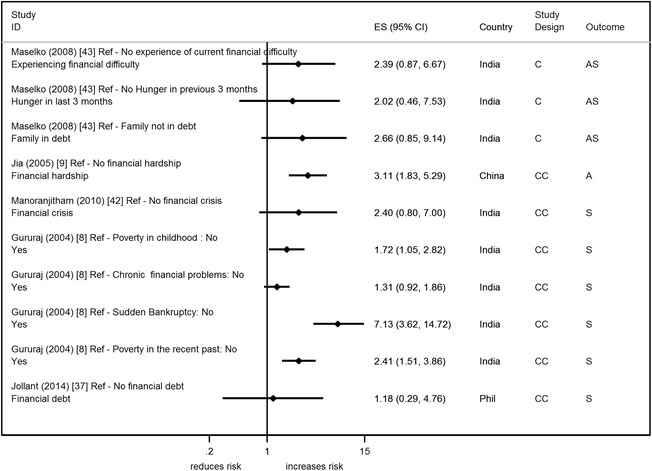
Forest plot of studies reporting on financial difficulty and suicide/attempted suicide risk. Country: Bang – Bangladesh; Indon – Indonesia; Pak –Pakistan; Phil – Philippines; SL – Sri Lanka; Thai – Thailand; Viet – Vietnam. Study design: C – Cohort; CC – Case–control; X – Cross-sectional. Outcome: A – Attempted suicide only; S – Suicide only; AS – Attempted suicide and suicide. ES: Cohort studies report estimates of relative risk (except for Maselko (2008) which reports odds ratios), and case–control/cross-sectional studies report odds ratios

Five studies reported on measures of financial difficulty (Fig. 10) [8, 9, 37, 42, 43]; some studies reported on more than one measure of financial difficulty [8, 43]. The majority reported measures of long-term/persistent financial difficulty, with only two studies reporting on acute financial problems [8, 42]. Four out of the ten associations (3 from India – same study [8], and 1 from China [9]) show statistical evidence of an increased risk of suicide and attempted suicide with financial difficulty, with OR ranging from 1.7 to 7.1. Two studies made adjustments in either the analysis or design, with only one providing the unadjusted and adjusted estimates (included SEP factors). This showed a reduction after adjustments were made [43]. Only one reasonable/high quality study found an increased odds of suicide with financial difficulty [42].

**Fig. 11:**
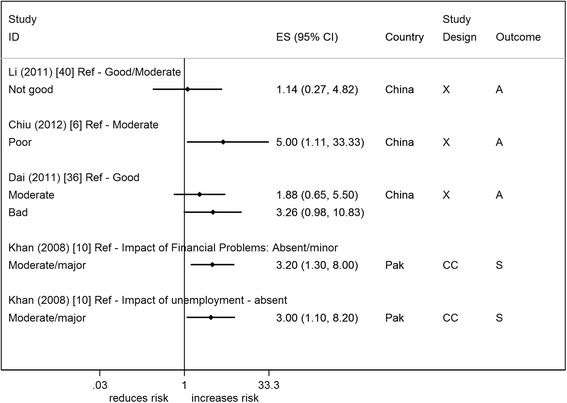
Forest plot of studies reporting on subjective measures of financial circumstance and suicide/attempted suicide risk. Country: Bang – Bangladesh; Indon – Indonesia; Pak –Pakistan; Phil – Philippines; SL – Sri Lanka; Thai – Thailand; Viet – Vietnam. Study design: C – Cohort; CC – Case–control; X – Cross-sectional. Outcome: A – Attempted suicide only; S – Suicide only; AS – Attempted suicide and suicide. ES: Cohort studies report estimates of relative risk (except for Maselko (2008) which reports odds ratios), and case–control/cross-sectional studies report odds ratios

Four studies reported on subjective measures of financial circumstance (self-rated measure) (Fig. 11) [6, 10, 36, 40] and they all showed a positive association of suicide/attempted suicide with a poorer perceived financial situation. There was statistical evidence to support this association in two studies (two measures from the same study) (OR range 3–5) [6, 10]. One study which showed the weakest evidence, a study from China conducted in an elderly population (≥60 years), made no adjustments for age or gender in their analysis [40]. All four studies were of reasonable/high quality.

#### Other measures of SEP

We included two further measures of SEP which are not conventionally considered to be measures of SEP; we believe that in this setting marital status and religion may provide additional insights into a person’s position in society. We provide the results of this analysis in the supplementary materials (see Additional file 2). Briefly, we found that most studies reported an increased risk of suicide/attempted suicide in those divorced/widowed/separated/remarried. None of the studies, however, looked at the interaction between marital status with age and/or gender. There were mixed findings in relation to religion; studies from China showed statistical evidence of a lower risk of suicide in people who were not religious, whereas studies from India [8, 42], Indonesia [11], and Sri Lanka [32] show an increased risk of suicide and attempted suicide in individuals who reported being religious.

#### Measures across studies

Several studies reported more than one measure of SEP. As previously shown, a great deal of heterogeneity exists between studies. In order to investigate whether this heterogeneity between studies and between SEP measures exists for measures within studies, we investigated the direction of the effects observed for each SEP measure within a study (see Additional file 2). It appears that even within studies, the direction of the association of different SEP measures with risk of suicide/attempted suicide varies. When restricted to the high/reasonable quality studies, the amount of heterogeneity decreased but there were still studies showing associations in opposite directions for different SEP measures.

## Discussion

This review included 31 studies from nine out of 18 LAMIC in South and South East Asia. Studies have included a wide range of measures of SEP and used cross-sectional (42 %), cohort (10 %) and case control (48 %) designs to investigate the association of SEP with suicide/attempted suicide risk. Most studies indicate that lower levels of SEP are associated with an increased risk of suicide/attempted suicide, though findings are not always consistent between and within countries. There does not seem to be an apparent difference in the direction or magnitude of the associations by outcome (suicide, attempts only, attempts and suicide). The SEP risk factors with the most consistent association across studies were asset based measures (particularly composite scores); education; measures of financial difficulty; and subjective measures of financial circumstances. Several of these studies show a greater than threefold increased risk with measures of SEP– with the largest and most consistent association with subjective measures of financial circumstance. The quality of the studies included in this review were of moderate to low quality, with over half the studies using self-reported outcomes. The majority of these associations are also likely to suffer from over-adjustment bias as other SEP measures were often adjusted for in the analysis.

We used studies included in our restricted analysis (including only reasonable/high quality studies) in order to inform our conclusions. Whilst this restriction resulted in more consistent results across studies, there were still some outliers. For example for asset based measures of SEP, the study by Chowdury et al. (2005) (cross-sectional) showed the weakest evidence (point estimate close to null) of an increased risk of the outcome with poorer assets, but this was the only study to report on self-reported attempted suicide [35]. The other two studies reported on objectively measured suicide deaths. A similar weak association was observed in one study in the restricted analysis of poorer subjective measures of financial circumstances [40]. This weak association maybe explained by the fact that this study was focused on an older population, who have been reported to have higher degrees of financial satisfaction [51], therefore making it more difficult to observe any differences between attempters and non-attempters. Associations of education level with the risk of the outcome consistently showed an increased risk of suicide/attempted suicide with lower levels of education; however, there was one case–control study which reported a point estimate consistent with a protective effect of being illiterate and death by suicide (no statistical evidence) [42].

Compared to asset based measures, education and financial measures, the association of suicide/attempted suicide with occupation and income were inconsistent in the reasonable/high quality studies. The studies showed an increased, decreased, and no associated risk. The reasons for this inconsistency may be due to varying definitions of exposure between studies, bias, the factors adjusted for, and/or the comparison categories used. It may also be possible that variation in the extent of social support/protection between countries might explain the inconsistencies observed [52], though this is unlikely to be the case because even within countries a large about heterogeneity exists. This large amount of variation between studies makes it difficult to draw a conclusion on these associations.

### Possible mechanisms

The findings of this review suggest that poorer levels of assets, subjective measures of financial circumstance and higher levels of financial difficulty increase the risk of suicide/attempted suicide. As suggested by other authors, it is possible that experiencing higher levels of financial adversity, both on a short term and long term basis, increases levels of anxiety, feelings of hopelessness, and emotional reactivity [14, 15]. Interestingly the findings of this review suggest that the largest and most consistent SEP measure associated with increased suicide/attempted suicide was with poorer levels of perceived financial status, suggesting that an individual’s subjective financial wellbeing may be a more important predictor of a suicide/attempted suicide than more conventional objective measures. It could be that with increasing urbanisation and globalisation, individuals are experiencing higher levels of relative deprivation due to rising social inequality [53]. This is where an individual’s status may not be considered poor when objectively measured, but when individuals compare themselves to others in their local and/or the global community (through migration and media exposure), they may feel higher levels of discontentment. This then can lead to increased levels of frustration, anxiety and depression. Equally, as subjective measures of financial circumstance were collected at the same time as outcome assessment, these associations may be due to reverse causation – individuals experiencing low mood maybe more likely to feel negatively about their financial situation.

### Comparison to other studies

To the best of our knowledge there has been no other published systematic review of individual level SEP and suicide/attempted suicide risk in LAMIC in South and South East Asia. There has, however, been a systematic review published on area level SEP and suicide risk [5]. This review also reported considerable heterogeneity between study findings. The authors concluded that there appeared to be an inverse association of suicide with higher area level SEP; the measures which were most likely to show an increased risk of suicide with lower levels of SEP, were measures of poverty or composite scores (asset based) [5]. This study excluded studies not written in English, included only suicide as an outcome and importantly only looked at area level associations – thus limiting its ability to make inferences about individual risk. In addition we were unable to draw comparisons with this review for LAMIC in Asia because the study did not present this information. However, its general finding that asset based measures of SEP were most frequently associated with an increased risk of suicide was replicated in the current review on an individual level in LAMIC in South and South East Asia.

In a selective review of risk factors for suicide in developing countries [4] lower levels of SEP were associated with increased suicide risk. These results were based on the findings from two studies, a Chinese study (which used injury deaths as the control group and was therefore excluded from this review) [20] and a review from India based on the elderly (≥60 years)[54]. The findings of the current review are also consistent with reviews of individual risk factors for suicide/attempted suicide which have been conducted primarily in non-LAMIC and mainly in a western context, though western studies are less likely to report on asset based measures or subjective measures of financial circumstance [3, 55].

Lund *et al.* (2010) conducted a systematic review looking at poverty and common mental disorders (excluding suicide/self-harm) in LAMIC [18]. The findings of this review were consistent with our findings in that they found a more consistent association between common mental disorders and lower levels of education, housing, social class, socio-economic status and financial stress. As with our review, associations with employment and income were more ambiguous.

### Strengths and limitations

This is the first review to systematically summarise what is currently known about the association of individual level SEP and suicide/attempted suicide in LAMIC in South and South East Asia. Whilst we did not perform a meta-analysis because of heterogeneity, the literature search, screening and data extraction were conducted to the same robust standards needed for quantitative synthesis. There are, however, several limitations to this review. Whilst we recognised that SEP is often recorded in studies but not always reported, and took steps to try and limit this publication bias, this review is still likely to suffer from this bias. We suspect that even though authors do not report on SEP in their publications, the likelihood is that they would still have measured it as part of their study and did not report on it because this was either not the focus of their study or the results were non-significant; it was beyond the scope of this review to contact all authors who did not report on SEP in the full text article. A further limitation of assessing the association between SEP and suicide/attempted suicide was that several studies presented only adjusted estimates, some of which were likely to be over adjusted for other SEP factors. SEP measures are proxy measures of the concept of SEP and therefore by definition are related to each other. Each SEP measure may contribute to the causal pathway between other SEP measures and suicide/attempted suicide risk and adjustment can bias the results towards the null [56]. In attempting to determine the effect of this adjustment we investigated this in the few studies which presented both crude and adjusted estimates. Lastly, we did not make any study exclusions based on study quality, but did conduct a restricted secondary analysis excluding studies with a poor overall quality score and used the results of this analysis to form our conclusions. This strategy, however, is limited because for some reasonable/high quality studies (as defined by the median score), the scoring may have resulted in a high score being achieved under one assessment criteria (e.g. selection) but very low scores for another criteria (e.g. outcome). Kumar *et al.* (2013) is an example of this, where this study scores 100 % under the selection criteria but scored 0 % in the exposure scale – giving an overall score of 70 % (reasonable/high quality).

## Conclusion

Based on this systematic review of published studies, there is evidence that lower SEP increases the likelihood of suicide/attempted suicide in LAMIC in South and South East Asia. Conventional measures of SEP, e.g. unemployment, show inconsistent findings across countries. Asset based measures (composite measures), education and measures of financial difficulty/perception appear to show a more consistent inverse association with suicide/attempted suicide. The findings are, however, severely limited by the quality of the study designs, analysis strategies and measures used. Larger, better quality studies in the general population looking at this association in these countries are needed. In particular, future studies in this area should make improvements to outcome assessment (e.g. hospital/coroner reports) and include a range of SEP measures. These improvements will be needed in order to draw useful public health conclusions.

## Additional files

Additional file 1:
**Supplementary methods.** Description of data: Detailed description of search strategy used, additional methods and adapted quality rating scales used. (DOC 112 kb) Additional file 2:
**Supplementary results.** Description of data: Detailed quality rating results; description of questions used to derive composite scores; results of marital status and religion; and summary of measures across studies. (DOCX 74 kb)

## Abbreviations

SEP: Socioeconomic position
LAMIC: Low and middle income countries
