# Supplementary material for: Association of socio-economic position and suicide/attempted suicide in low and middle income countries in South and South-East Asia – a systematic review
Source: BMC Public Health. 2015 Oct 15;15:1055. doi: 10.1186/s12889-015-2301-5 (PMC4608117; doi:10.1186/s12889-015-2301-5)
Supplement: Additional file 1: — Supplementary methods. Description of data: Detailed description of search strategy used, additional methods and adapted quality rating scales used. (DOC 112 kb) [file 12889_2015_2301_MOESM1_ESM.doc]

# Supplementary methods

### Search strategies used to identify papers for inclusion in the systematic review

**Medline search**:

1. Socioeconomic Factors/ or Education/ or Occupations/ or unemployment/ or employment/ or Income/ or Marital Status/ or Marriage/ or divorce/ or Religion/ or Poverty/ or Culture/ or ethnology/ or bankruptcy/ or economics/ or crowding/ or toilet facilities/ or water supply/
2. (socioeconomic or socio economic or socio-economic or caste or education* or Occupation or Employ* or Unemploy* or Income or Job or Money or Social* or Marital or Marriage or divorce or Religio* or Poverty or Finance or Wealth or Culture or ethnolog* or Deprivation or bankrupt* or economic or debt or SES or SEP or rural or urban or (car adj2 ownership) or (vehicle adj2 ownership) or (motor* adj2 ownership) or (bike adj2 ownership) or household construction or household material or (cooking adj2 fuel) or electricity supply).mp.
3. 1 or 2
4. suicide/ or suicidal ideation/ or self-injurious behavior/ or self mutilation/ or near drowning/ or drowning/ or suicide, attempted/ or overdose/ or burns/ or firearms/
5. (selfharm* or self-harm* or self harm* or suicid* or self-injur* or self injur* or selfinjur* or self mutilat* or self-mutilat* or selfmutilat* or self inflict* or self-inflict* or selfinflict* or parasuicide or self-destruct* or self destruct* or selfdestruct* or self-abuse or selfabuse or self abuse or self-poison* or self poison* or selfpoison* or drown* or self immolation or self-immolation or immolation or hanging or burning or jumping or firearms or self-hit* or self hit* or selfhit* or head-banging or head banging).mp.
6. 4 or 5
7. Developing countries/ or asia/ or Afghanistan/ or Bangladesh/ or Bhutan/ or Cambodia/ or China/ or India/ or Indonesia/ or Korea/ or Lao/ or Malaysia/ or Maldives/ or Mongolia/ or Myanmar/ or Nepal/ or Pakistan/ or Philippines/ or Sri Lanka/ or Thailand/ or Vietnam/
8. (developing countr* or asia or low income count* or middle income countr* or Afghanistan or Bangladesh or Bhutan or Cambodia or China or India or Indonesia or Korea or Lao or Malaysia or Maldives or Mongolia or Myanmar or Nepal or Pakistan or Philippines or Sri Lanka or Ceylon or Thailand or Vietnam or Timor-Leste or Timor Leste or TimorLeste or east timor or east-timor or easttimor or west timor or west-timor or westtimor or Tibet or Burma).mp.
9. 7 or 8
10. 3 and 6 and 9
11. (biomass or syncope or waste management or ecology or (burn and infection) or fuel or Vietnam War or ("u.s." and miliarty) or (us and military) or (us and soldiers) or ("u.s." and soldiers) or infection# or burn management or prevention of burn* or bed nets or mosquito* or malaria).mp.
12. syncope/ or waste management/ or Genes, Transgenic, Suicide/ or Malaria/
13. 10 not (11 or 12)
14. (animals not (humans and animals)).sh.
15. (meta analysis or "review" or "scientific integrity review").pt.
16. (qualitative not quantitative).ti
17. pollut*.mp.
18. 13 not (or/ 14-17)

**Medline in Process**:

1. (socioeconomic or socio economic or socio-economic or caste or education* or Occupation or Employ* or Unemploy* or Income or Job or Money or Social* or Marital or Marriage or divorce or Religio* or Poverty or Finance or Wealth or Culture or ethnolog* or Deprivation or bankrupt* or economic or debt or SES or SEP or rural or urban or (car adj2 ownership) or (vehicle adj2 ownership) or (motor* adj2 ownership) or (bike adj2 ownership) or household construction or household material or (cooking adj2 fuel) or electricity supply).mp.
2. (selfharm* or self-harm* or self harm* or suicid* or self-injur* or self injur* or selfinjur* or self mutilat* or self-mutilat* or selfmutilat* or self inflict* or self-inflict* or selfinflict* or parasuicide or self-destruct* or self destruct* or selfdestruct* or self-abuse or selfabuse or self abuse or self-poison* or self poison* or selfpoison* or drown* or self immolation or self-immolation or immolation or hanging or burning or jumping or firearms or self-hit* or self hit* or selfhit* or head-banging or head banging).mp.
3. (developing countr* or asia or low income count* or middle income countr* or Afghanistan or Bangladesh or Bhutan or Cambodia or China or India or Indonesia or Korea or Lao or Malaysia or Maldives or Mongolia or Myanmar or Nepal or Pakistan or Philippines or Sri Lanka or Ceylon or Thailand or Vietnam or Timor-Leste or Timor Leste or TimorLeste or east timor or east-timor or easttimor or west timor or west-timor or westtimor or Tibet or Burma).mp.
4. (biomass or syncope or waste management or ecology or (burn and infection) or fuel or Vietnam War or ("u.s." and miliarty) or (us and military) or (us and soldiers) or ("u.s." and soldiers) or infection# or burn management or prevention of burn* or bed nets or mosquito* or malaria).mp.
5. (animals not (humans and animals)).sh.
6. (meta analysis or "review" or "scientific integrity review").pt.
7. (qualitative not quantitative).ti.
8. pollut*.mp.
9. 1 and 2 and 3
10. 9 not (or/5-8)

**PsychInfo search**:

1. exp Socioeconomic Status/ or exp Family Socioeconomic Level/ or exp "Culture (Anthropological)" or exp ethnology/ or exp Educational Background/ or exp education/ or exp Income level/ or exp lower income level/ or exp middle income level/ or upper income level/ or exp caste system/ or exp occupations/ or exp employment status/ or exp unemployment/ or exp marital status or exp marital satisfaction/ or exp marital relation/ or exp marital conflict/ or exp marriage/ or exp religion/ or exp religious beliefs/ or exp poverty/ or exp poverty areas/ or exp finance/ or exp money/ or exp Deprivation/ or exp divorce/ or financial strain/ or economics/ or crowding/
2. (socioeconomic or socio economic or socio-economic or caste or education* or Occupation or Employ* or Unemploy* or Income or Job or Money or Social* or Marital or Marriage or divorce or Religio* or Poverty or Finance or Wealth or Culture or ethnolog* or Deprivation or bankrupt* or economic or debt or SES or SEP or rural or urban or (car adj2 ownership) or (vehicle adj2 ownership) or (motor* adj2 ownership) or (bike adj2 ownership) or household construction or household material or (cooking adj2 fuel) or electricity supply).mp.
3. 1 or 2
4. exp suicide/ or exp attempted suicide/ or exp suicidal ideation/ or exp self injurious behavior/ or exp self destructive behavior/ or exp self inflicted wounds/ or exp self mutilation/ or exp drug overdoses/ or exp head-banging/
5. (selfharm* or self-harm* or self harm* or suicid* or self-injur* or self injur* or selfinjur* or self mutilat* or self-mutilat* or selfmutilat* or self inflict* or self-inflict* or selfinflict* or parasuicide or self-destruct* or self destruct* or selfdestruct* or self-abuse or selfabuse or self abuse or self-poison* or self poison* or selfpoison* or drown* or self immolation or self-immolation or immolation or hanging or burning or jumping or firearms).mp.
6. 4 or 5
7. Exp Developing countries/ or exp Asians/
8. (developing countr* or asia or low income count* or middle income countr* or Afghanistan or Bangladesh or Bhutan or Cambodia or China or India or Indonesia or Korea or Lao or Malaysia or Maldives or Mongolia or Myanmar or Nepal or Pakistan or Philippines or Sri Lanka or Ceylon or Thailand or Vietnam or Timor-Leste or Timor Leste or TimorLeste or east timor or east-timor or easttimor or west timor or west-timor or westtimor or Tibet or Burma).mp.
9. 7 or 8
10. 3 and 6 and 9
11. (biomass or syncope or waste management or ecology or (burn and infection) or fuel or Vietnam War or ("u.s." and miliarty) or (us and military) or (us and soldiers) or ("u.s." and soldiers) or infection#).mp
12. (animal not ((human or inpatient or outpatient) and animal)).po.
13. (systematic review or review).ti
14. immigrant.mp. or exp Immigration/
15. 10 not (or/ 11-14)
16. (qualitative not quantitative).ti
17. pollut*.mp.
18. 15 not (16 or 17)

**Embase search:**

1. Socioeconomics/ or Education/ or Occupations/ or exp unemployment/ or exp employment/ or Lowest income group/ or marriage/ or divorce/ or Religion/ or Poverty/ or economics/ or ethnology/ or cultural anthropology/ or sanitation/ or water supply/ or crowding/
2. (socioeconomic or socio economic or socio-economic or caste or education* or Occupation or Employ* or Unemploy* or Income or Job or Money or Social* or Marital or Marriage or divorce or Religio* or Poverty or Finance or Wealth or Culture or ethnolog* or Deprivation or bankrupt* or economic or debt or SES or SEP or rural or urban or (car adj2 ownership) or (vehicle adj2 ownership) or (motor* adj2 ownership) or (bike adj2 ownership) or household construction or household material or (cooking adj2 fuel) or electricity supply).mp.
3. 1 or 2
4. Suicide/ or Suicide attempt/ or suicidal behaviour/ or suicidal ideation/ or automutilation/ or near drowning/ or drowning/ or hanging/ or jumping/ or firearm/
5. (selfharm* or self-harm* or self harm* or suicid* or self-injur* or self injur* or selfinjur* or self mutilat* or self-mutilat* or selfmutilat* or self inflict* or self-inflict* or selfinflict* or parasuicide or self-destruct* or self destruct* or selfdestruct* or self-abuse or selfabuse or self abuse or self-poison* or self poison* or selfpoison* or drown* or self immolation or self-immolation or immolation or hanging or burning or jumping or firearms or self-hit* or self hit* or selfhit* or head-banging or head banging).mp.
6. 4 or 5
7. Developing country/ or Asia/ or South Asia/ or Southeast Asia/ or Afghanistan/ or Bangladesh/ or Bhutan/ or Cambodia/ or China/ or India/ or Indonesia/ or Korea/ or Lao/ or Malaysia/ or Maldives/ or Mongolia/ or Myanmar/ or Nepal/ or Pakistan/ or Philippines/ or Sri Lanka/ or Thailand/ or Vietnam/
8. (developing countr* or asia or low income count* or middle income countr* or Afghanistan or Bangladesh or Bhutan or Cambodia or China or India or Indonesia or Korea or Lao or Malaysia or Maldives or Mongolia or Myanmar or Nepal or Pakistan or Philippines or Sri Lanka or Ceylon or Thailand or Vietnam or Timor-Leste or Timor Leste or TimorLeste or east timor or east-timor or easttimor or west timor or west-timor or westtimor or Tibet or Burma).mp.
9. 7 or 8
10. (biomass or syncope or waste management or ecology or (burn and infection) or fuel or Vietnam War or ("u.s." and miliarty) or (us and military) or (us and soldiers) or ("u.s." and soldiers) or infection#).mp
11. Faintness/ or waste management/ or suicide gene therapy/
12. ((animal or nonhuman) not (human and (animal or nonhuman))).de.
13. ("book review" or "conference proceeding review" or "journal conference review" or "journal review" or "report review" or "review").pt.
14. 3 and 6 and 9
15. 14 not (or/10-13)
16. Flooding/
17. 15 not 16

**Web of Science Search:**

1. TS=(socioeconomic OR socio-economic OR caste OR education* OR Occupation OR Employ* OR Unemploy* OR Income OR Job OR Money OR Social* OR Marital OR Marriage OR divorce OR Religion OR Poverty OR Finance OR Wealth OR Culture OR ethnolog* OR Deprivation OR bankrupt* OR economic OR debt OR SES OR SEP) AND TS=(selfharm* OR self-harm* OR self harm* OR suicid* OR self-injur* OR self injur* OR selfinjur* OR self mutilat* OR self-mutilat* OR selfmutilat* OR self inflict* OR self-inflict* OR selfinflict* OR parasuicide OR self-destruct* OR self destruct* OR Selfdestruct* OR self-abuse OR selfabuse OR self abuse OR self-poison* OR self poison* OR selfpoison* OR drown* OR self immolation OR self-immolation OR immolation OR hanging OR burning OR jumping OR firearms) AND TS=(developing countr* OR asia OR low income count* OR middle income countr* OR Afghanistan OR Bangladesh OR Bhutan OR Cambodia OR China OR India OR Indonesia OR Korea OR Lao OR Malaysia OR Maldives OR Mongolia OR Myanmar OR Nepal OR Pakistan OR Philippines OR Sri Lanka OR Ceylon OR Thailand OR Vietnam OR Timor-Leste OR Timor Leste OR TimorLeste OR east timor OR east-timor OR easttimor OR west timor OR west-timor OR westtimor OR Tibet OR Burma)
2. TS=(Ecology OR Biomass or greenhouse or sustain* or pollut* or aerosols or trees or "Vietnam War" or "Climate Change" or "Childhood drowning" or "US Military" or "U.S. Military" or "U.S. Soldiers" or "US Soldiers" or "Burn Wound" or "Burn Management" or "U.S. Service" or "US Service" or "waste management")
3. TI=(review or systematic review)
4. #1 not (#2 or #3)

##

### Further methodological details

*Data extraction*

Where numerical measures of association were not presented these were calculated from the raw figures (if provided). It was not possible to calculate odds ratios for matched case control studies using a matched analysis, because the paired data were not presented. In order to compare across studies the estimates calculated/extracted compared the lowest SEP category to the highest. In instances where there was more than one SEP category (e.g. education achievement – did not attend school; primary schooling; secondary schooling) and the estimates presented use the lowest category as the base category (e.g. not attended), we only used the inverted estimate of the highest vs. the lowest category (e.g. secondary vs. not attended) in the review. In order to compare results across studies, we calculated effect estimates (where possible) from raw numbers using a similar base group as a comparison; as the main aim of this review was to get a consensus of the direction of the effect across studies, this calculated effect estimate took precedence over estimates adjusted for possible confounding factors.

*Analysis*

There was substantial heterogeneity between studies for different SEP measures. In order to determine whether the heterogeneity observed was due to differences in what was adjusted or matched for in each study, we created three fields to indicate whether a study was adjusted for the following:

1. Age and/or gender
2. SEP factors - at least one, but could include several. As area of residence or region is likely to be related to area level SEP, we categorised studies which adjusted or matched for area as having adjusted for SEP.
3. Other factors – a range of other factors were adjusted for. These included mental illness.

We only categorised a matched study as having adjusted for the matching criteria if an appropriate matched analysis was undertaken.

##

Table 1 - Categorisation of SEP measures reported by included studies

| **SEP Major Grouping** | **SEP Minor Grouping** | **SEP Measure** |
| --- | --- | --- |
| Asset based measures | Composite measure | Wealth score |
|  |  | Asset score |
|  | Asset ownership/access | Motorbike ownership |
|  |  | TV ownership |
|  |  | Land ownership |
|  |  | Access of electricity |
|  |  | Access to latrine/toilet |
|  |  | Access to mobile phone |
|  | Household construction | Materials used for wall |
|  |  | Materials used for roof |
|  |  | Materials used for floor |
|  |  | Materials used for whole house |
| Education | Education level | Number of years |
|  |  | Highest qualification |
| Occupation | Unemployment | Unemployment |
|  | Occupation type | Occupation type* |
| Financial Measures | Income | Family income |
|  |  | Monthly income |
|  | Financial difficulty | Family debt |
|  |  | Financial crisis |
|  |  | Financial debt |
|  |  | Financial hardship |
|  |  | Financial problems |
|  |  | Impact of financial problems |
|  |  | Sudden bankruptcy |
|  |  | Hunger in last 3 months |
|  |  | Poverty |
|  | Subjective measures of financial circumstance | Subjective measures of financial circumstance (self-rated) |
| Other measures | Marital status | Marital status |
|  | Religious status | Religious vs. no religious |
|  | Religion | Religious group |

*several different categories dependent on study

### Newcastle-Ottawa quality assessment scale

**CASE CONTROL STUDIES**

**Selection**

1) Is the case definition adequate?

SUICIDE

1. yes, (an acceptable definition is used – coroner defined/police confirmed/standardised verbal autopsy) *****
2. yes, e.g. record linkage or based on self-reports
3. no description

ATTEMPTED SUICIDE (max two stars)

1. yes, Independent validation (e.g. hospital records)******
2. yes, Self-report with more than one question (e.g. asks about attempt but also method chosen etc)******
3. yes, Self-report –only one question asked*****
4. no description

2) Representativeness of the cases

1. consecutive or obviously representative series of cases *****
2. potential for selection biases or not stated

3) Selection of Controls

1. community controls (non-family/non-neighbourhood (e.g. same street)) *****
2. other controls
3. no description

4) Definition of Controls (only relevant for non-fatal attempts)

1. no history of disease (endpoint) *****
2. not relevant – suicide *****
3. no description of source

**Comparability**

5) Comparability of cases and controls on the basis of the design or analysis

1. study controls for gender *****
2. study controls for age *****

**Exposure**

6) Ascertainment of exposure

1. structured interview where blind to case/control status or written self-report*****
2. interview not blinded to case/control status
3. no description

7) Same method of ascertainment for cases and controls

1. yes (e.g. if informants used for cases, then should also be used for controls) *****
2. no/ not known

8) Non-Response rate

1. Assessment made and response rate is not socially patterned by case/control status (can be written statement)*****
2. Response rate is similar in cases and controls but no indication if socially patterned
3. rate different and no designation
4. Not described

**COHORT STUDIES**

**Selection**

1) Representativeness of the exposed cohort

1. truly representative of the general population in the community (includes everyone in an area or shows that the sample chosen is representative of the larger community)*****
2. selected group e.g. farmers, doctors
3. no description

2) Ascertainment of exposure

1. clear description of how exposure was measured and categorised *****
2. no or inadequate description

3) Demonstration that outcome of interest was not present at start of study

1. yes *****
2. not relevant – suicide *****
3. no

**Comparability**

4) Comparability of cohorts on the basis of the design or analysis

1. study controls for gender*****
2. study controls for age *****

**Outcome**

5) Assessment of outcome

1. independent blind (to exposure) assessment *****
2. record linkage *****
3. self report
4. no description

6) Adequacy of follow up of cohorts

1. complete follow up - all subjects accounted for *****
2. subjects lost to follow up unlikely to introduce bias - small number lost - > 85% follow up, or description provided of those lost and this is not socially patterned *****
3. follow up rate < 85% and no description of those lost
4. no statement

**CROSS SECTIONAL STUDIES[[1]](#footnote-2)**

**Selection:**

1) Representativeness of the sample:

a) Truly representative of the general population. * (all subjects or random sampling)

b) Somewhat representative of the average in the target population. * (non-random sampling)

c) Selected group of users.

d) No description of the sampling strategy.

2) Non-respondents(max 2 stars)

1. response rate >50% *****
2. response rate is not socially patterned*****
3. response rate is unsatisfactory <50%, or the comparability between respondents and non-respondents is unsatisfactory.
4. No description of the response rate or the characteristics of the responders and the non-responders.

3) Ascertainment of the exposure (risk factor):

1. clear description of how exposure was measured and categorised *****
2. no or inadequate description

**Comparability:**

4) The subjects in different outcome groups are comparable, based on the study design or analysis. Confounding factors are controlled.

a) The study controls for gender*

b) The study control for age *

**Outcome:**

5) Assessment of the outcome:

1. Record linkage. *
2. yes, Self-report with more than one question (e.g. asks about attempt but also method chosen etc)*****
3. Self-report
4. No description.

1. Adapted from an adapted version used in: “Are Healthcare Workers’ Intentions to Vaccinate Related to their Knowledge, Beliefs and Attitudes? A Systematic Review”. [↑](#footnote-ref-2)
