# Supplementary material for: Association of socio-economic position and suicide/attempted suicide in low and middle income countries in South and South-East Asia – a systematic review
Source: BMC Public Health. 2015 Oct 15;15:1055. doi: 10.1186/s12889-015-2301-5 (PMC4608117; doi:10.1186/s12889-015-2301-5)
Supplement: Additional file 2: — Supplementary results. Description of data: Detailed quality rating results; description of questions used to derive composite scores; results of marital status and religion; and summary of measures across studies. (DOCX 74 kb) [file 12889_2015_2301_MOESM2_ESM.docx]

# Supplementary Results

### Results of quality rating

| **Cohort Studies** | |  |  |  |
| --- | --- | --- | --- | --- |
|  |  |  |  |  |
|  |  | Sauvaget (2009) [45] | Rebholz (2011) [44] | Maselko (2009) [43] |
| **Selection** | |  |  |  |
| 1) Representativeness of the exposed cohort | |  |  |  |
|  | a) truly representative of the general population in the community (includes everyone in an area or shows that the sample chosen is representative of the larger community)* |  |  | * |
|  | b) selected group eg farmers, doctors | * | * |  |
|  | c) no description |  |  |  |
| 2) Ascertainment of exposure | |  |  |  |
|  | a)      clear description of how exposure was measured and categorised * | * | * | * |
|  | b)      no or inadequate description |  |  |  |
|  |  |  |  |  |
| 3) Demonstration that outcome of interest was not present at start of study | |  |  |  |
|  | a)      yes * |  |  |  |
|  | b)      not relevant – suicide * | * | * |  |
|  | c)      no |  |  | * |
| **Comparability** | |  |  |  |
| 4) Comparability of cohorts on the basis of the design or analysis | |  |  |  |
|  | a)      study controls for gender* | * | * |  |
|  | b)      study controls for age * | * | * |  |
|  |  |  |  |  |
| **Outcome** | |  |  |  |
| 5) Assessment of outcome | |  |  |  |
|  | a)      independent blind (to exposure) assessment * |  |  |  |
|  | b)      record linkage * | * | * |  |
|  | c)      self report |  |  | * |
|  | d)      no description |  |  |  |
|  |  |  |  |  |
| 6) Adequacy of follow up of cohorts | |  |  |  |
|  | a)      complete follow up - all subjects accounted for * |  |  |  |
|  | b)      subjects lost to follow up unlikely to introduce bias - small number lost - > 85% follow up, or description provided of those lost and this is not socially patterned * |  | * | * |
|  | c)      follow up rate < 85% and no description of those lost |  |  |  |
|  | d)      no statement | * |  |  |

| Cross-sectional Studies | |  |  |  |  |  |  |  |  |  |  |  |
| --- | --- | --- | --- | --- | --- | --- | --- | --- | --- | --- | --- | --- |
|  |  | Lee (2007) [39] | Ma (2009) [12] | Chiu (2012) [6] | Dai (2011) [36] | Blum (2012) [30] | Feroz (2012) [7] | Ma (2010) [41] | Sun (2010) [47] | Chowdhury (2005) [35] | Li (2011) [40] | Devries (2011) [31] |
| **Selection** | |  |  |  |  |  |  |  |  |  |  |  |
| 1) Representativeness of the sample: | |  |  |  |  |  |  |  |  |  |  |  |
|  | a) Truly representative of the general population. * (all subjects or random sampling) |  | * |  |  |  | * | * | * | * | * |  |
|  | b) Somewhat representative of the average in the target population. * (non-random sampling) |  |  | * |  |  |  |  |  |  |  |  |
|  | c) Selected group of users. | * |  |  | * | * |  |  |  |  |  | * |
|  | d) No description of the sampling strategy. |  |  |  |  |  |  |  |  |  |  |  |
|  |  |  |  |  |  |  |  |  |  |  |  |  |
| 2) Non-respondents(max 2 stars) | |  |  |  |  |  |  |  |  |  |  |  |
|  | a)      response rate >50% * |  | * | * |  |  | * | * | * | * | * | * |
|  | b)      response rate is not socially patterned* |  |  |  | * |  |  |  |  |  |  |  |
|  | c)      response rate is unsatisfactory <50%, or the comparability between respondents and non-respondents is unsatisfactory. |  |  |  |  |  |  |  |  |  |  |  |
|  | d)      No description of the response rate or the characteristics of the responders and the non-responders. | * |  |  |  | * |  |  |  |  |  |  |
|  |  |  |  |  |  |  |  |  |  |  |  |  |
| 3) Ascertainment of the exposure (risk factor): | |  |  |  |  |  |  |  |  |  |  |  |
|  | a)      clear description of how exposure was measured and categorised * |  | * | * | * | * | * | * |  | * | * | * |
|  | b)      no or inadequate description | * |  |  |  |  |  |  | * |  |  |  |
|  |  |  |  |  |  |  |  |  |  |  |  |  |
| **Comparability:** | |  |  |  |  |  |  |  |  |  |  |  |
| 4) The subjects in different outcome groups are comparable, based on the study design or analysis. Confounding factors are controlled. | |  |  |  |  |  |  |  |  |  |  |  |
|  | a) The study controls for gender* | * |  | * | * | * |  | * | * |  |  | * |
|  | b) The study control for age * | * |  | * | * | * |  |  | * |  |  | * |
|  |  |  |  |  |  |  |  |  |  |  |  |  |
| **Outcome:** | |  |  |  |  |  |  |  |  |  |  |  |
| 5) Assessment of the outcome: | |  |  |  |  |  |  |  |  |  |  |  |
|  | a)      Record linkage. * |  |  |  |  |  |  |  |  |  |  |  |
|  | b)      yes, Self-report with more than one question (e.g. asks about attempt but also method chosen etc)* |  |  |  |  |  |  |  |  |  |  |  |
|  | c)      Self report |  |  |  | * |  |  | * | * | * | * |  |
|  | d)      No description. | * | * | * |  | * | * |  |  |  |  | * |

##

| Case Control Studies | |  |  |  |  |  |  |  |  |  |  |
| --- | --- | --- | --- | --- | --- | --- | --- | --- | --- | --- | --- |
|  | | Manoranjitham (2010) [42] | Kurihara (2009) [11] | Gururaj (2004) [8] | Sisask (2010) [32] | Zhang (2010) [13] | Jollant (2014) [37] | Sun (2014) [46] | Jia (2005) [9] | Vijayakumar (1999) [49] | Khan (2008) [10] |
| **Selection** | |  |  |  |  |  |  |  |  |  |  |
| 1) Is the case definition adequate? | |  |  |  |  |  |  |  |  |  |  |
|  | SUICIDE |  |  |  |  |  |  |  |  |  |  |
|  | a)      yes, (an acceptable definition is used – coroner defined/police confirmed/standardised verbal autopsy) ** | * | * | * |  | * |  | * |  | * | * |
|  | b)  yes, eg record linkage or based on self reports |  |  |  |  |  | * |  |  |  |  |
|  | c)      no description |  |  |  |  |  |  |  |  |  |  |
| ATTEMPTED SUICIDE (max two stars) | |  |  |  |  |  |  |  |  |  |  |
|  | a)  yes, Independent validation (e.g. hospital records)** |  |  |  | * |  |  |  | * |  |  |
|  | b)  yes, Self-report with more than one question (e.g. asks about attempt but also method chosen etc)** |  |  |  |  |  |  |  |  |  |  |
|  | c)  yes, Self-report –only one question asked* |  |  |  |  |  |  |  |  |  |  |
|  | d)  no description |  |  |  |  |  |  |  |  |  |  |
| 2) Representativeness of the cases | |  |  |  |  |  |  |  |  |  |  |
|  | a)      consecutive or obviously representative series of cases * | * | * | * |  | * |  | * |  | * | * |
|  | b)      potential for selection biases or not stated |  |  |  | * |  | * |  | * |  |  |
| 3) Selection of Controls | |  |  |  |  |  |  |  |  |  |  |
|  | a)      community controls (non-family/non-neighbourhood (e.g. same street)) * |  |  |  | * | * | * |  |  |  |  |
|  | b)      other controls | * | * | * |  |  |  | * | * | * | * |
|  | c)      no description |  |  |  |  |  |  |  |  |  |  |
| 4) Definition of Controls (only relevant for non-fatal attempts) | |  |  |  |  |  |  |  |  |  |  |
|  | a)      no history of disease (endpoint) * |  |  |  | * |  |  |  |  |  |  |
|  | b)      not relevant – suicide * | * | * | * |  | * | * | * |  | * | * |
|  | c)      no description of source |  |  |  |  |  |  |  | * |  |  |
| **Comparability** | |  |  |  |  |  |  |  |  |  |  |
| 5) Comparability of cases and controls on the basis of the design or analysis | |  |  |  |  |  |  |  |  |  |  |
|  | a)      study controls for gender * | * | * | * | * | * | * | * | * | * | * |
|  | b)      study controls for age * | * | * | * | * | * | * | * | * | * | * |
|  |  |  |  |  |  |  |  |  |  |  |  |
| **Exposure** | |  |  |  |  |  |  |  |  |  |  |
| 6) Ascertainment of exposure | |  |  |  |  |  |  |  |  |  |  |
|  | a)      structured interview where blind to case/control status or written self-report* |  |  |  |  |  |  |  | * |  |  |
|  | b)      interview not blinded to case/control status | * | * | * | * | * | * | * |  | * | * |
|  | c)      no description |  |  |  |  |  |  |  |  |  |  |
| 7) Same method of ascertainment for cases and controls | |  |  |  |  |  |  |  |  |  |  |
|  | a)      yes (e.g. if informants used for cases, then should also be used for controls) * | * | * |  | * | * | * | * | * | * | * |
|  | b)      no/ not known |  |  | * |  |  |  |  |  |  |  |
| 8) Non-Response rate | |  |  |  |  |  |  |  |  |  |  |
|  | a)      Assessment made and response rate is not socially patterned by case/control status (can be written statement)* |  | * |  |  |  |  |  |  |  |  |
|  | b)      Response rate is similar in cases and controls but no indication if socially patterned |  |  |  |  | * |  | * |  | * |  |
|  | c)      rate different and no designation | * |  |  |  |  |  |  |  |  |  |
|  | d)      Not described |  |  | * | * |  | * |  | * |  | * |

### Questions used to derive composite measures and factors adjusted for in analysis

| **Study** | **Questions used to derive aggregate measure** | | **Factors adjusted for in analysis** |
| --- | --- | --- | --- |
| Khan (2008) [10] | No. of rooms in house | | Age |
|  | Toilet facilities | | Gender |
|  | Ownership of: | | Area of residence |
|  |  | Land |  |
|  |  | Vehicle (pedal bike/motor bike/car) |  |
|  |  | Fan/Iron/Washing machine |  |
|  |  | Radio/ Cassette player |  |
|  |  | Refrigerator/Freezer |  |
|  |  | Video player/TV |  |
|  |  | Air conditioner |  |
|  | Household income | |  |
|  | Type of education given to children | |  |
|  | Education of head of household | |  |
|  | Occupation of head of household | |  |
|  | Area of residence | |  |
| Blum (2012) [30] - China (A) & Vietnam (B) | Ownership of: | | Age |
|  |  | Clock/watch | Gender |
|  |  | Bicycle | Education level |
|  |  | Radio/TV | Family structure |
|  |  | Sewing machine | Employment/education status |
|  |  | Motorcycle/scooter | Migrant status |
|  |  | Refrigerator | Family history of suicide |
|  |  | Car | Quality of maternal/paternal relationship |
|  |  | Land | Cigarette use |
|  | Household condition | | Alcohol use |
|  |  | Drinking water source |  |
|  |  | Toilet facilities |  |
|  |  | Fuel/lighting source |  |
|  |  | No. of rooms |  |
|  |  | Quality of construction |  |
| Feroz (2012) [7] |  | Earning capacity |  |
|  |  | Housing status - quality of building |  |
|  |  | Possession of essential and luxury goods |  |
| Sauvaget (2009) [45] | Household assets | | Sex  Age  Randomisation group  Education level  Religion  Occupation  Type of house  Number of residents  Income  Chewing habits  Smoking habits  Drinking habits  Medical history |

**Other measures of SEP**

*Marital status*

Marital status was the second most commonly reported exposure in this review (19 studies) (Supplementary figure 1and Supplementary figure 2) [6, 8, 10-13, 31, 36, 37, 39-43, 46, 47, 49, 50]. Just under half of the studies controlled for other factors (including SEP) in their analysis or design [6, 10, 13, 31, 36, 39, 42]. Roughly equal numbers of studies reported an increased and decreased risk of suicide/attempted suicide with being unmarried, though only 5 of these studies show statistical evidence of an increased risk [6, 13, 40, 46] or reduced risk[31]. The one study from Thailand (city) which observed a statistical evidence of a reduction in attempted suicide was based on a female only sample. None of the studies investigated the interaction between marital status with age and/or gender. The majority of studies investigating other marital statuses indicated that compared to being married (differing definitions) the risk of suicide/attempted suicide was greater in those divorced/widowed/separated/remarried (Supplementary figure 2). Only one study indicated a reduction in risk of those single or widowed [47].

*Religion*

We identified ten estimates of the association of suicide/attempted suicide with not being religious (Supplementary figure 3) [8, 10, 11, 13, 32, 42, 46, 50]. The studies which show a decreased risk of suicide with not being religious were all from China [13, 46, 50]. The other studies included from other parts of South and South East Asia suggest an increased risk of suicide/attempted suicide with having low religious beliefs/ not being religious, or no association [ 8, 10, 11, 32, 42]. In the sensitivity analysis the majority of studies are consistent with an increased risk of suicide/attempted suicide with no/low religious beliefs [10, 32, 42]. However, one Chinese study remained in the restricted analysis which showed statistical evidence of a reduction in odds [13].

We included studies that looked at a person’s religious faith/grouping to determine whether being part of a minority religious group increases the risk of suicide/attempted suicide. Therefore for the purpose of this review, we have extracted/calculated the effect estimates using the most predominant religious group of the country as the comparison – 5 studies reported on this exposure (Supplementary figure 4) [7, 10, 43, 45, 48]. Studies included suggested both an increased and decreased risk of suicide/attempted suicide with minority religious faiths. The sensitivity analysis suggests that the reduction in risk observed, may be due to adjustments made to the analysis.

Supplementary figure 1- Forest plot of studies reporting on being unmarried

Country: Bang – Bangladesh; Indon – Indonesia; Pak –Pakistan; Phil – Philippines; SL – Sri Lanka; Thai – Thailand; Viet – Vietnam

Study design: C – Cohort; CC – Case-Control; X – Cross-sectional

Outcome: A – Attempted suicide only; S – Suicide only; AS – Attempted suicide and suicide

ES: Cohort studies report estimates of relative risk (except for Maselko (2008) which reports odds ratios), and case-control/cross-sectional studies report odds ratios.

Supplementary figure 2 - Forest plot of studies reporting on marital status

Country: Bang – Bangladesh; Indon – Indonesia; Pak –Pakistan; Phil – Philippines; SL – Sri Lanka; Thai – Thailand; Viet – Vietnam

Study design: C – Cohort; CC – Case-Control; X – Cross-sectional

Outcome: A – Attempted suicide only; S – Suicide only; AS – Attempted suicide and suicide

ES: Cohort studies report estimates of relative risk (except for Maselko (2008) which reports odds ratios), and case-control/cross-sectional studies report odds ratios.

Supplementary figure 3- Forest plot of studies reporting on being religious

Country: Bang – Bangladesh; Indon – Indonesia; Pak –Pakistan; Phil – Philippines; SL – Sri Lanka; Thai – Thailand; Viet – Vietnam

Study design: C – Cohort; CC – Case-Control; X – Cross-sectional

Outcome: A – Attempted suicide only; S – Suicide only; AS – Attempted suicide and suicide

ES: Cohort studies report estimates of relative risk (except for Maselko (2008) which reports odds ratios), and case-control/cross-sectional studies report odds ratios.

Supplementary figure 4- Forest plot of studies reporting on religious faith (comparison is majority religious faith in the country)

Country: Bang – Bangladesh; Indon – Indonesia; Pak –Pakistan; Phil – Philippines; SL – Sri Lanka; Thai – Thailand; Viet – Vietnam

Study design: C – Cohort; CC – Case-Control; X – Cross-sectional

Outcome: A – Attempted suicide only; S – Suicide only; AS – Attempted suicide and suicide

ES: Cohort studies report estimates of relative risk (except for Maselko (2008) which reports odds ratios), and case-control/cross-sectional studies report odds ratios.

### Measures across studies

|  | **Study** | Feroz (2012) [7] | Lee (2007) [39] | Ma (2009) [12] | Sun (2010) [47] | Ma (2010) [41] | Dai (2011) [36] | Li (2011) [40] | Chiu (2012) [6] | Blum (2012) A [30] | Rebholz (2011) [44] | Chowdury (2005) [35] | Maselko (2008) [43] | Sauvaget (2009) [45] | Devries (2011) A [31] | Devries (2011) B [31] | Blum (2012) B [30] | Zhang (2004) [50] | Jia (2005) [9] | Zhang (2010) [13] | Manoranjitham (2010) [42] | Sun (2014) [46] | Vijayakumar (1999) [49] | Gururaj (2004) [8] | Sisask (2010) A [32] | Kulkarni (2011) [38] | Kumar (2013) [48] | Kurihara (2009) [11] | Khan (2008) [10] | Jollant (2014) [37] | Sisask (2010) B [32] | Sisask (2010) C [32] |
| --- | --- | --- | --- | --- | --- | --- | --- | --- | --- | --- | --- | --- | --- | --- | --- | --- | --- | --- | --- | --- | --- | --- | --- | --- | --- | --- | --- | --- | --- | --- | --- | --- |
| **Asset-based** | **Composite SEP** | ↑ |  |  |  |  |  |  |  | ↑ |  |  |  | ↑ |  |  | ↑ |  |  |  |  |  |  |  |  |  |  |  | ↑ |  |  |  |
|  | **Access to services** | ↑ |  |  |  |  |  |  |  |  |  |  |  |  |  |  |  |  |  |  |  |  |  |  |  |  |  |  |  |  |  |  |
|  | **Household construction** | ↑ |  |  |  |  |  |  |  |  |  |  |  | ↑ |  |  |  |  |  |  |  |  |  |  |  |  |  |  |  |  |  |  |
|  | **Vehicle ownership** | ↑ |  |  |  |  |  |  |  |  |  |  |  |  |  |  |  |  |  |  |  |  |  |  |  |  |  |  |  |  |  |  |
|  | **Land Ownership** | ↑↓ |  |  |  |  |  |  |  |  |  | x |  |  |  |  |  |  |  |  |  |  |  |  |  |  |  |  |  |  |  |  |
|  | **TV Ownership** | ↑ |  |  |  |  |  |  |  |  |  |  |  |  |  |  |  |  |  |  |  |  |  |  |  |  |  |  |  |  |  |  |
| **Education** | | ↓ | ↑ |  | ↑ |  | ↑ |  |  | ↑ | ↑ |  | ↑ | ↑↓ |  |  | ↑ | ↑ | ↑ | ↑ | ↓ |  | ↓ |  |  | ↑ | ↑ | ↑ | ↑ | ↑ |  |  |
| **Occupation** | **Unemployment** |  | ↓ |  |  |  |  |  |  | ↓ | ↑ | ↑ |  | ↑ |  |  | x | ↑ |  | ↑ | ↓ |  |  | ↑ |  |  | ↓ | ↑ | ↑ |  |  |  |
|  | **Occupation groups** | ↓ |  |  |  |  | x |  |  |  | ↑ |  | ↓ | ↑ |  |  |  |  |  |  |  |  |  |  |  |  |  |  |  |  |  |  |
| **Financial measures** | **Income** | ↑ |  | ↑ | ↓ |  |  |  |  |  |  |  | ↑ | x |  |  |  | ↑ | ↑ | ↑ | x |  | ↓ |  |  |  |  |  |  |  |  |  |
|  | **Financial difficulty** |  |  |  |  |  |  |  |  |  |  |  | ↑ |  |  |  |  |  | ↑ |  | ↑ |  |  | ↑ |  |  |  |  | ↑ | ↑ |  |  |
|  | **Financial perception** |  |  |  |  |  | ↑ | ↑ | ↑ |  |  |  |  |  |  |  |  |  |  |  |  |  |  |  |  |  |  |  | ↑ |  |  |  |
| **Other measures** | **Unmarried** |  | ↑ |  | ↓ |  | ↑ | ↑ | ↑ |  |  |  | ↓ |  | ↓ | ↓ |  | ↓ |  | ↑ |  | ↑ | ↑ |  |  |  |  |  |  |  |  |  |
|  | **Other marital status** |  |  | ↑ | ↑↓ | ↑ | ↑ |  |  |  |  |  |  |  | ↑ | ↑ |  |  |  | ↑ | ↑ |  |  | ↑ |  |  |  | ↑ |  |  |  |  |
|  | **Religion** |  |  |  |  |  |  |  |  |  |  |  |  |  |  |  |  | ↓ | ↓ |  | ↑ | ↓ |  | ↑ | ↑ |  |  | ↑ | ↑ |  | ↑ | x |
|  | **Religious faith** | ↑ |  |  |  |  |  |  |  |  |  |  | ↑ | ↓ |  |  |  |  |  |  |  |  |  |  |  |  | ↓ |  | ↓x |  |  |  |
|  |  |  |  |  |  |  |  |  |  |  |  |  |  |  |  |  |  |  |  |  |  |  |  |  |  |  |  |  |  |  |  |  |
| ↑ - Increased risk with lower SEP | | | | | ↓ - Decreased risk with lower SEP | | | | | | | X – Direction of association uncertain | | | | | | | | Statistically significant result | | | | | | |  |  |  |  |  |  |
